# The lipid-lowering effects of fenugreek gum, hawthorn pectin, and burdock inulin

**DOI:** 10.3389/fnut.2023.1149094

**Published:** 2023-03-23

**Authors:** Yi Wang, Yu Zheng, Yi Liu, Guoshun Shan, Baojie Zhang, Qian Cai, Jiayue Lou, Yang Qu

**Affiliations:** School of Pharmacy, Liaoning University of Traditional Chinese Medicine, Dalian, Liaoning, China

**Keywords:** Galactomannan, pectin, inulin, polysaccharide, hyperlipidemia, intestinal bacteria

## Abstract

**Objective:**

The present study aimed to investigate the lipid-lowering effects and mechanisms of fenugreek gum (FG), hawthorn pectin (HP), and burdock inulin (BI) on high-fat diet (HFD)-induced hyperlipidemic rats.

**Methods:**

In this study, high-fat diet (HFD) together with fat emulsion administration were used to establish hyperlipidemia model. The biochemical indices were assayed after administration of FG, HP, and BI. Their effects were evaluated by factor analysis. Alterations of gut microbiota and short chain fatty acids (SCFAs) in the cecal were assessed to illustrate the mechanism of lipid lowering.

**Results:**

The supplementation of FG, HP, and BI on HFD-fed rats decreased the levels of serum lipid and reduced the HFD-related liver and testicle damage. In the scatter plot of factor analysis, HP and BI were closer to normal fat diet (NFD) group in restoring the severity of hyperlipidemia, while FG and HP enhanced the excretion of cholesterol and bile acids (BAs). The levels of total SCFAs, especially butyric acid reduced by HFD were increased by HP. The ratio of Firmicutes to Bacteroidetes increased by HFD was reduced by HP and BI. FG, HP, and BI enriched intestinal probiotics, which were related to bile acid excretion or lipid-lowering.

**Conclusions:**

FG inhibited the absorption of cholesterol and enhanced the excretion of it, as well as increased the abundance of beneficial bacteria. While BI restored the imbalance of intestinal microbiota. HP enhanced the excretion of cholesterol and BAs, and restored the imbalance of intestinal microbiota. It was also utilized by intestinal microorganisms to yield SCFAs. This study suggested that FG, HP, and BI possessed the potential to be utilized as dietary supplements for obesity management.

## 1. Introduction

About 41.9% of adults in China suffer from hyperlipidemia, with a higher prevalence in men (1). Currently, statins are the most commonly used drugs for treating hyperlipidemia. However, statin treatment has adverse effects such as abnormal liver function, muscle pain, diabetes risk, and abnormal cognitive function (2). Therefore, researchers are working to find more effective treatments for hyperlipidemia with fewer adverse effects.

Galactomannan, pectin, and inulin, as typical representatives of dietary polysaccharides, were widely used in the daily diets (3). Dietary fiber has been used for long periods to maintain health and to prevent or treat a range of common diseases, including diabetes, hyperlipidemia, intestinal diseases, and obesity (4).

Fenugreek gum (FG), hawthorn pectin (HP), and burdock inulin (BI) were the main polysaccharides component in fenugreek seeds (*Trigonella foenum-graecum* L.), hawthorn fruits (*Crataegus* spp) and burdock roots (*Arctium lappa* L.). Galactomannan composed 89.5% of FG (5). HP was acidic polysaccharides composed of galacturonic acid skeleton, with Ara, Glc, and Gal on the side chains (6). And BI was fructan, which belonged to the neutral oligosaccharide (7). All of them were applied as food additives. FG can be used as a thickening, stabilizing, and emulsifying agents in food products to improve their quality and shelf life by reducing the staling and retrogradation rates (8, 9). And HP was also used as a thickening agent (10). Inulin was applied as a substitute for fat and sucrose in icecream to enhance consistency and stickiness (11).

Early studies have shown that FG, HP, and BI had anti-hyperlipidemia activity (10, 12, 13). They were fermented by the intestinal bacteria *in vivo* to yield short-chain fatty acids (SCFAs), which showed beneficial effects on the body, and they improved the composition of intestinal bacteria conversely (14–16). Hyperlipidemia was accompanied with disorders of intestinal bacteria, thus maintaining the balance of which was an important way for the lipid-lowering effect of drugs or functional food (14, 17). Besides, polysaccharides sequestrated with bile acids, influence the bioaccessibility of cholesterol to lower the level of cholesterol (18). An early study showed that a supplement of 15% FG in the daily diet led to reductions in weight gain and total serum cholesterol in normal male C57BL/6J mice. FG decreased the ratio of Firmicutes to Bacteroidetes (F/B). The TC absorption was lowered, and bile acid excretion was elevated by oral infused HP at the dosage of 300 mg/kg/d in high-cholesterol diet (HCD) hamsters (13).The supplement of 1% BI in the daily diet led to the increase in the levels of propionic acid and butyric acid in caecal contents in high-fat diet (HFD)-mice (16). Therefore, FG, HP, and BI were applied in the study to compare their effects on HFD rats. Lipid metabolism index, SCFAs, and the composition of intestinal bacteria were assayed. Factor analysis was used to evaluate their effects and explore their mechanism of lipid-lowering. Furthermore, correlation analysis was used to find the relationship between intestinal bacteria and lipid metabolism parameters. The results of this research tended to reveal the different mechanism of the lipid-lowering effects of FG, HP, and BI.

## 2. Materials and methods

### 2.1. Materials

HFD was purchased from Shengmin Scientific Research Animal Farm Nanjing, China. The fat emulsion composed of 65.5% pure water, 10% lard, 2.5% cholesterol, 5% sucrose, 5% fructose, 5.5% Tween 80, and 6.5% glycerol was concocted for laboratory use. Total cholesterol (TC) (batch no. 20210702), triglyceride (TG) (batch no. 20210703), low-density lipoprotein cholesterol (LDL-C) (batch no. 20210702), high-density lipoprotein cholesterol (HDL-C) (batch no. 20210702), alanine aminotransferase (ALT) (batch no. 20210715), aspartate aminotransferase (AST) (batch no. 20210716), total bile acids (TBA) (batch no. 20210928), and total protein (TP) (batch no. 20211019) assay kits were purchased from Jiancheng Biological Engineering Institute, Nanjing, China. Cyclic adenosine monophosphate (cAMP) (batch no. 21071535N), cyclic guanosine monophosphate (cGMP) (batch no. 1071536N), and testosterone (T) (batch no. 21071530N) ELISA kits were purchased from Kexing Biological Engineering Institute, Shanghai, China. The standards of dextran with molecular weights of 5,000, 11,600, 23,800, 48,600, 80,900, 148,000, 273,000, 409,800, and 667,800 Da (batch nos. 102084138, 102136543, 102124529, 102104509, 102108375, 102089360, 102110878, 102124507, 102104510) were purchased from Sigma Biological company, Germany. The standards of dextran with molecular weights of 1,150,000, 1,740,000, and 2,457,000 Da (batch nos. D1150K, D1740K, D2400K) were purchased from American Polymer Standards Corporation, American. All other chemical reagents used in this work were commercially available and of analytical or chromatographic grade. Fenugreek seed, hawthorn fruits, and burdock roots were purchased from a local market in Dalian, and identified by professor Tianmin Wang, school of Pharmacy, Liaoning University of Traditional Chinese Medicine, as the *Trigonella foenum-graecum* L., *Crataegus pinnatifida* Bge., *Arctium lappa* L.

### 2.2. The preparation and chemical characterizations of FG, HP, and BI

#### 2.2.1. The preparation of FG, HP, and BI

The powders of fenugreek seeds, hawthorn fruits, and burdock roots were added to distilled water at a ratio of 1:20 (w:v) and extracted at 95°C for 60 min two times. The extracts were combined and concentrated to one-third of the original volume, then added two times the volume of 95% ethanol. The precipitates were collected after resting overnight at 4°C (7, 19, 20). They were formulated into an aqueous solution of 25 mg/mL.

#### 2.2.2. The chemical characterization of FG, HP, and BI

The content of the total polysaccharide was determined by UV–vis spectrophotometry according to the method reported in the literature (21). Briefly, 1 mL of the sample solution was added to 1 mL of 5% phenol solution. Subsequently, 5 mL of concentrated sulfuric acid was added to the reaction mixture. After 1 h, the absorbance was measured at 490 nm along with the reference standard, and the results were expressed as glucose equivalents. The linear range of the standard sample was 0.05 ~ 0.10 mg/mL, calibration curve: *y* = 10.119x–0.0208, *R*^2^ = 0.9935.

The content of total protein was determined by UV–vis spectrophotometry according to the method reported in the literature (22). Briefly, 1 mL of the sample solution was added to 5 mL of Coomassie brilliant blue reagent (composed of Coomassie brilliant blue 0.01% G250, 8.5% phosphoric acid and 5% ethanol). After 2 min, the absorbance was measured at 595 nm along with the reference standard, and the results were expressed as bovine serum albumin (BSA) equivalents. The linear range of the standard sample was 0.0217 ~ 0.1194 mg/mL, calibration curve: *y* = 3.6928x + 0.1089, *R*^2^ = 0.9967.

Molecular weights of FG, HP, and BI were determined employing high performance liquid chromatography with a Shimadzu LC-10A HPLC system outfitted with a BRT105-104-102 (8 × 300 mm) gel chromatography columns and a Shimadzu refractive index detector (RID) with 0.05 M NaCl solution as the mobile phase at a flow rate of 0.6 mL/min (40°C). The molecular weights of FG, HP, and BI were calculated based on the standard curve of a succession of molecular weights standards. The chemical composition and molecular weight of FG, HP, and BI are shown in Table 1.

**Table 1:** The chemical compositions of FG, HP, and BI.

	FG	HP	BI
Recovery rate (%)	1.62	10.04	9.60
Total saccharides (%)	39.60 ± 0.04	43.28 ± 0.02	40.28 ± 0.01
Protein (%)	11.13 ± 0.08	1.29 ± 0.01	1.44 ± 0.02
Molecular weight (Mw) (×10^3^Da)	2,970	182.0	3.019

(Mean ± SD, *n* = 3).

### 2.3. The hypolipidemic effects of FG, HP, and BI

#### 2.3.1. Animals and diets

In this study, all animal experiments complied with the ARRIVE guidelines and were carried out in accordance with the National Research Council’s Guide for the Care and Use of Laboratory Animals. Ethical approval for the involvement of animals in this study was granted by Liaoning University of Traditional Chinese Medicine Research Ethics Committee, license number 210000420210205, 4/17/2021.

Thirty male Sprague–Dawley (SD) rats (200 ~ 250 g) were purchased from Changsheng Biotechnology Co., Ltd. Liaoning, China (Permit number: SCXK (Liao) 2020–0001). All animal procedures in this experiment were strictly handled in accordance with the regulation for the use and care of laboratory animals. The rats were reared in a large room with constant temperature (25 ± 1°C) and humidity (55 ± 5%) with 12 h day/night cycle, and were free to obtain food and water.

After 1 week of adaptation, the rats were randomly divided into the following five groups: the normal-fat diet (NFD) group, the HFD group, the FG group, the HP group, and the BI group, and each group was assigned six rats. Rats in the NFD group were given maintenance diets, and rats in the HFD, FG, HP, and BI groups were given HFD together with 2 mL fat emulsion per day for 6 weeks. During the last 2 weeks of modeling, FG, HP, and BI groups were given FG, HP, and BI aqueous solutions by gavage at a dose of 250 mg/kg per day, respectively.

#### 2.3.2. Blood and tissue sample collection

After the 6-week experiment, all experimental rats fasted overnight. Then, they were anesthetized using ethyl carbamate at a dose of 1,200 mg/kg (i.p.) and sacrificed. Whole blood samples were immediately collected from the abdominal aorta for the detection of biochemical indexes. The feces and cecal contents were collected, weighed, frozen immediately in liquid nitrogen, and stored at −80°C. The fresh liver, spleen, thymus, and testicles of all rats were removed and weighed. The organ index was calculated as follows:


$$Organindex\left(\%\right)=\frac{Organweight}{Bodyweight}$$

#### 2.3.3. Determination of serum and fecal biochemical indices

The blood samples were placed in plastic centrifuge tubes at 37°C for 30 min and centrifuged at 900 g for 10 min to obtain the serum samples. Then, biochemical indicators of the serum, including TC, TG, LDL-C, HDL-C, ALT, AST, cAMP, cGMP, and T were immediately analyzed by the kits according to the manufacturer’s instructions. In addition, fecal samples were added anhydrous ethanol solution at a ratio of 1:9 (w:v), and ground with a tissue masher at 10,000 g to make 10% tissue homogenate (23). Contents of fecal TC, TBA, and TP were measured using assay kits following the manufacturer’s instructions.

#### 2.3.4. Histopathological analysis

The livers of each rat were dissected, washed with saline, and fixed with 10% formalin solution for 24 h. Subsequently, tissue cutting and hematoxylin and eosin (H&E) staining were performed by Cairong Ming, Department of Pathology, Liaoning University of Traditional Chinese Medicine. Images were captured using a microscope at 200× magnification.

#### 2.3.5. Analysis of short-chain fatty acids (SCFAs)

Cecal contents were added 50 μL 0.2% H_3_PO_4_ solution containing 4-methylvaleric acid as internal standard (0.668 mg/mL), quickly sealed for headspace injection on Agilent 7890B-5977B GC–MS instrument. Headspace injection conditions: Headspace sampler vial heat temperature: 80°C; loop heat temperature: 140°C; transfer line heat temperature: 160°C; GC cycle time: 30 min; heat time: 20 min; equilibration time: 10 min; pressurization time: 0.15 min; injection time: 0.5 min. Chromatographic conditions: The SCFAs have chromatographically separated on DB-WAX (DB-1MS) capillary column (30 m × 0.25 mm, 0.25 μm, Agilent Corporation, United States). Injection mode: splitless; inlet temperature 250°C; ion source temperature 230°C; transfer line temperature 250°C, quadrupole temperature 150°C. Temperature programming was set as follows: the initial temperature was 60°C, then rose to 120°C at 30°C/min, followed with 5°C/min to 140°C, and maintained for 1 min, then rose to 150°C in 1 min and to 160°C in 1 min. Finally, it rose to 230°C at 35°C/min, and the post-operation temperature was maintained at 230°C for 5 min. The carrier gas was helium at a flow rate of 1.0 mL/min. MS conditions: Electron bombardment ion source (EI), electron energy 70ev, solvent delay 4.5 min, scan mode was full scan and selected ion monitor (SIM) mode, scan range *m/z* 30–200.

#### 2.3.6. High-throughput sequencing analysis of intestinal bacteria

The bacterial DNA was extracted from cecal contents samples with a QIAamp Fast DNA stool Mini Kit (Qiagen, Cat# 51604), and PCR amplification was conducted with barcoded specific bacterial primers targeting the variable region 3–4 (V3–V4) of the 16S rRNA gene: forward primer 338F: 5′- ACTCCTACGGGAGGCAGCA-3′ and reverse primer 806R: 5′-GGACTACHVGGGTWTCTAAT-3′ (24). Construction of sequencing libraries and paired-end sequencing was performed on an Illumina NovaSeq6000 platform at Biomarker Technologies Co, Ltd. (Beijing, China) according to standard protocols. Paired-end reads were merged using FLASH v1.2.7 (25), and tags with more than six mismatches were discarded. The merged tags with an average quality score < 20 in a 50 bps sliding window were determined using Trimmomatic (26), and those shorter than 350 bps were removed. Possible chimeras were further removed, and the denoised sequences were clustered into operational taxonomic units (OTUs) with 97% similarity using USEARCH (version 10.0). Taxonomy was assigned to all OTUs by searching against the Silva database (Release128) using QIIME software. The microbial diversity was further analyzed using the difference between the samples, and the significance test was conducted with linear discriminate analysis effect size (LEfSe). To assess the effects of FG, HP, and BI on the composition of intestinal microbiota, V3–V4 regions of 16S rDNA were sequenced through HTS technology based on the Illumina Hiseq 2,500 sequencing platform (Biomarker Technologies Corporation, Beijing, China). The analyses of microbiota were conducted on BMK Cloud platform.^1^

### 2.4. Statistical analysis

#### 2.4.1. Statistical analysis for biochemical indices

The experimental data were expressed as mean ± standard deviation, and SPSS 26.0 statistical software was used for data analysis. The differences among groups were analyzed using one-way analysis of variance (one-way ANOVA). If the variances were equal, LSD tests were applied, and if the variances were not equal, Tamhane’s T2 tests were applied for *post hoc* tests. If the normal distributions were not satisfied, Mann–Whitney *U* tests were applied for *post hoc* tests. *P* < 0.05 was considered statistically significant.

#### 2.4.2. Factor analysis for biochemical indices

Factor analysis based on principal component analysis was applied to biochemical indices, which were significantly differences between groups by SPSS 26.0 statistical software. Bartlett’s test and KMO measure were used to measure the reasonableness of the test results. As a general rule, the factors with an eigenvalue greater than 1.0 were retained based on Scree Plots. The correlation coefficients were analyzed by principal component analysis and subsequent rotation according to the standard varimax criterion. The correlation between parameters was attributed to their common dependence on independent entities called ‘factors.’ The coefficients that linked the parameters to factors were named “factor loading.” Then, the factor scores were calculated based on ‘factor loading,’ and Origin software was used to draw a 3D scatter plot of the factor scores of each group.

#### 2.4.3. Statistical analysis for intestinal microbiome

Alpha diversity analysis was used to study the species richness, evenness, and sequencing depth in specific environments. Beta diversity analysis was used to study the species diversity among different environmental communities. The Metastats test was applied to detect features that were significantly different between assigned taxa. Linear discriminant analysis (LDA) and effect size (LEfSe) analysis were performed to identify the dimensional intestinal bacteria and characterize the microbial differences between different treatment groups. The LDA was used to quantify the effect size of each feature. A significance alpha value of less than 0.05 and an effect size threshold of 3.5 were used for this analysis.

#### 2.4.4. Correlation analysis for intestinal bacteria and biochemical indices

Correlation analysis between intestinal bacteria and biochemical indices was conducted by Spearman correlation on BMKCloud platform (see text footnote 1).

## 3. Results

### 3.1. Effects of FG, HP, and BI on body weight and organ index

**Figure 1:**
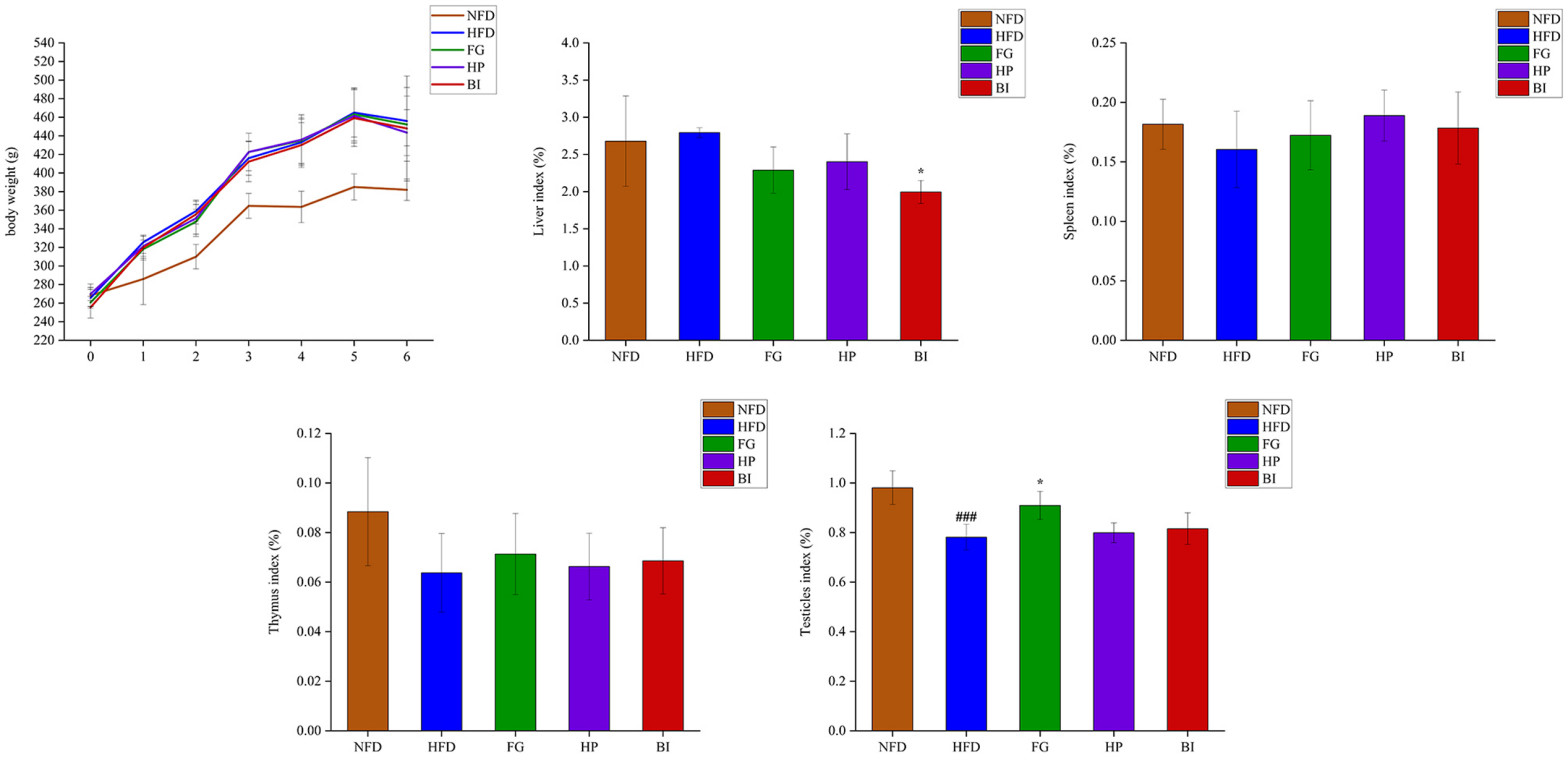
Effects of FG, HP, and BI treatment on body-weight and organ index on HFD-fed rats. The body weight, liver index, spleen index, thymus index, testicles index were shown, Values were expressed as mean ± SD in each group (*n* = 6). Significantly different from NFD group, ###*p* < 0.001. Significantly different from HFD group, *p < 0.05.

As shown in (Figure 1), HFD-feeding for 6 weeks induced a significant growth in body weight compared with the NFD group. The effects of FG, HP, and BI on weight gain induced by HFD feeding were insignificant. The HFD-fed rats presented a significant decrease in the testicle index compared with the NFD-fed rats (*p* < 0.001), and FG supplementation significantly increased the testicle index (*p* < 0.05). HFD-feeding did not generate significant effects on the liver, spleen, and thymus index indices in rats. However, BI supplementation significantly decreased the liver index (*p* < 0.05).

### 3.2. Effects of FG, HP, and BI on serum biochemical parameters

**Figure 2:**
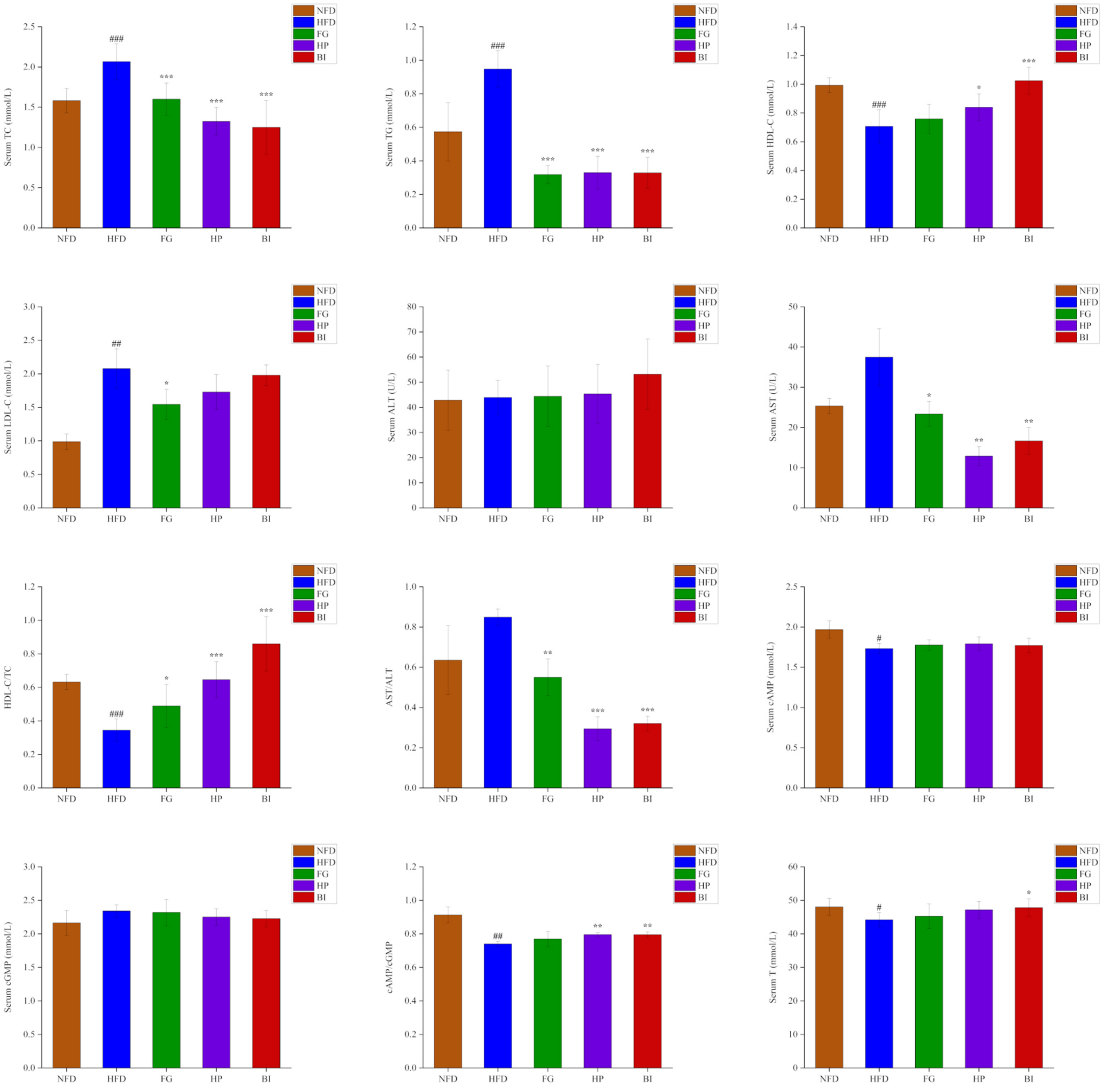
Effects of FG, HP, and BI treatment on serum biochemical indicators in HFD-fed rats. The Serum TC, Serum TG, Serum HDL-C, Serum LDL-C, Serum ALT, Serum AST, HDL-C/TC, AST/ALT, Serum cAMP, Serum cGMP, cAMP/cGMP, and Serum T levels were shown. Values were expressed as mean ± SD in each group (*n* = 6). Significantly different from NFD group, #*p* < 0.05, ##*p* < 0.01, ###*p* < 0.001. Significantly different from HFD group, *p < 0.05, **p < 0.01, ***p < 0.001.

As shown in (Figure 2), after the 6-week experiment, the serum levels of TC, TG, LDL-C in the HFD group significantly increased, and HDL-C, HDL-C/TC, cAMP, cAMP/cGMP, T significantly decreased compared with the NFD group (*p* < 0.05). Oral administration of FG, HP and BI significantly decreased the serum levels of TC, TG, and AST levels of HFD-fed rats (*p* < 0.05). In addition, FG, HP, and BI significantly increased HDL-C/TC and decreased AST/ALT values (*p* < 0.05), HP and BI significantly increased HDL-C level and cAMP/cGMP value (*p* < 0.05), and FG significantly decreased LDL-C level (*p* < 0.05), and BI significantly increased T level in hyperlipidemia rats (*p* < 0.05).

### 3.3. Effects of FG, HP and BI on fecal lipid and SCFAs levels

**Figure 3:**
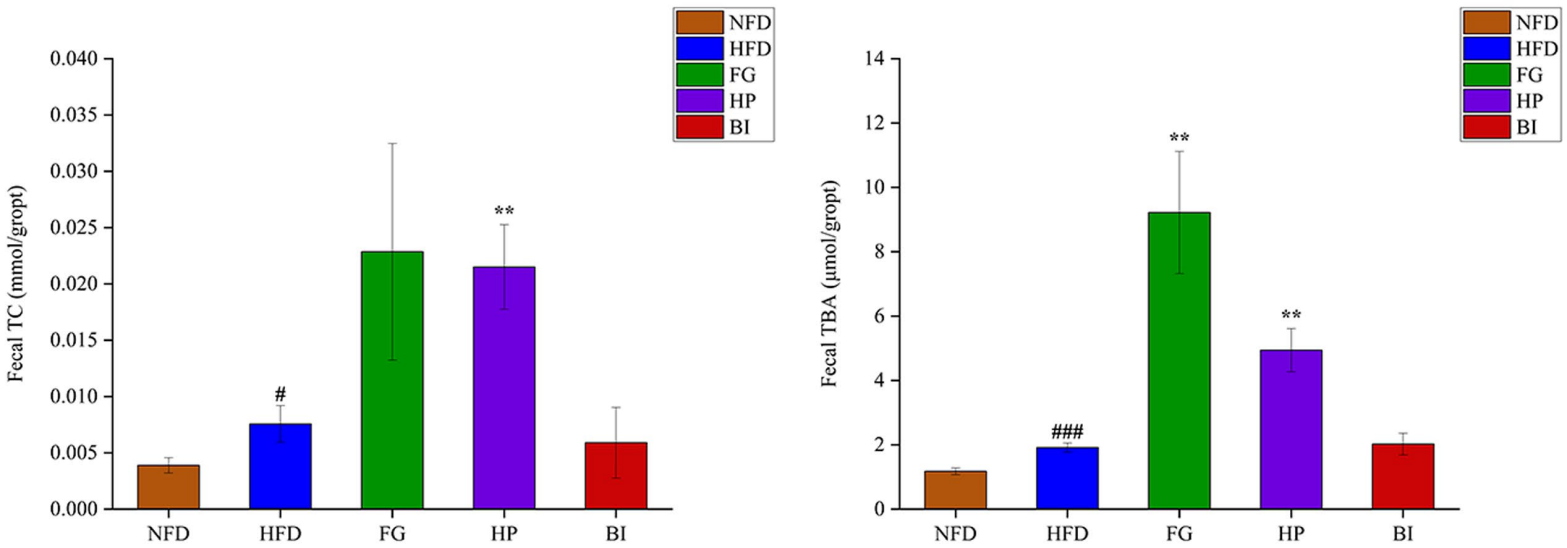
Effects of FG, HP and BI on the fecal lipid levels in HFD-fed rats. The Fecal TC and Fecal TBA levels were shown. Values are expressed as mean ± SD in each group (*n* = 6). Significantly different from NFD group, #*p* < 0.05, ###*p* < 0.001. Significantly different from HFD group, **p < 0.01.

As shown in (Figure 3), the results showed that HFD induced significantly increased levels of TC and TBA in the feces of rats. Furthermore, the daily intervention of FG significantly increased the content of TBA in feces, and HP significantly increased both the contents of fecal TC and TBA compared with the HFD group, which indicated that the lipid excretion in feces was enhanced. However, BI had no significant effect on the excretion of cholesterol and bile acids in hyperlipidemic rats. Acetic acid, propionic acid, butyric acid, isobutyric acid, and isovaleric acid in cecal contents were detected and quantified through GC–MS. As shown in Table 2, compared with the NFD group, high-fat diet supplementation significantly decreased the levels of SCFAs. While HP significantly increased the levels of SCFAs. HFD feeding resulted in significantly decreased levels of acetic acid, propionic acid, butyric acid, and isovaleric acid in the rat cecum (*p* < 0.05). HP significantly increased butyric acid levels in the HFD rats (*p* < 0.05). It also clearly increased the levels of cecal isobutyric acid and isovaleric acid (*p* < 0.05). BI significantly decreased the level of cecal propionic acid (*p* < 0.05). However, FG had no significant effect on the levels of SCFAs in the cecum of HFD rats.

**Table 2:** The levels each kind of SCFAs of NFD, HFD, FG, HP, and BI groups.

	NFD	HFD	FG	HP	BI
Acetic acid	903.74 ± 129.52	576.66 ± 153.47##	498.77 ± 121.87	628.01 ± 95.68	505.18 ± 55.04
Propionic acid	34.60 ± 2.40	27.74 ± 3.66#	24.60 ± 3.92	30.67 ± 1.60	22.52 ± 4.83*
Isobutyric acid	94.34 ± 19.96	132.12 ± 52.91	170.62 ± 45.18	202.25 ± 58.91*	123.15 ± 41.26
Butyric acid	390.36 ± 63.88	251.91 ± 110.23#	287.09 ± 102.55	420.08 ± 120.19*	194.36 ± 83.01
Isovaleric acid	4.64 ± 1.91	9.79 ± 4.87#	11.99 ± 3.23	14.25 ± 3.85*	10.03 ± 2.37
Total SCFAs	1427.68 ± 134.52	998.21 ± 81.70###	993.07 ± 188.37	1295.87 ± 237.87**	855.25 ± 134.54

(μg/g, mean ± SD, *n* = 6). Significantly different from NFD group, #*p* < 0.05, ##*p* < 0.01, ###*p* < 0.001. Significantly different from HFD group, *p < 0.05, **p < 0.01.

### 3.4. Factor analysis using biochemical indices as variables

**Figure 4:**
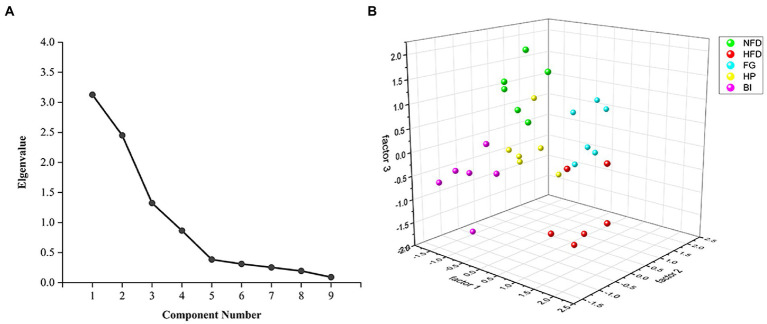
Factor analysis of biochemical indexes. **(A)** Scree plot depicting the eigenvalues of the factors extracted by factor analysis using biochemical indices as variables. **(B)** Scatter plot of FG, HP, and BI on the effects of hyperlipidemia related factors based on factor analysis.

In this study, the levels of TC, TG, HDL-C, LDL-C, AST, cAMP and T in serum, and TC, TBA in feces were applied to factor analysis. The KMO measure of sampling adequacy was 0.614, which was higher than the acceptance criterion of 0.5. The eigenvalues of the 9 factors were depicted in a scree plot in (Figure 4A). This criterion indicated that three factors should be extracted in the present study. The first, second, and third factors accounted for 34.734, 27.220, and 14.710% of the total variability, respectively. These three primary factors could explain 76.664% of the total variability in the dataset. The results of the factor analysis are shown in Table 3. According to this criterion, factor 1 could be labeled as “The seriousness of hyperlipidemia” because the selected biochemical variables, including serum TC, TG, HDL-C, and AST were related to the development of hyperlipidemia. Factor 2 was largely dependent on fecal TC and TBA, indicating the excretion of cholesterol. And factor 3 was largely dependent on LDL-C and cAMP, indicating the transportation of cholesterol and mobilization of fat. These three statistically obtained factors were used as the axes to show the distribution pattern of different study groups in the plot, as depicted in (Figure 4B) (27). The spots of FG, HP, and BI clustered well, suggesting fewer differences within the group. On factor 1, HFD greatly promoted the development of hyperlipidemia, while FG, HP, and BI restored this effect, and the spots of HP and BI were closer to those of NFD than those of FG, indicating a better effect. On factor 2, both FG and HP promoted the excretion of cholesterol. And on factor 3, FG, HP, and BI restored the effect on the transportation of cholesterol and fat mobilization by HFD.

**Table 3:** Rotated component matrix of factor 1, factor 2, and factor 3.

	Factor 1	Factor 2	Factor 3
Serum TC	0.871	0.005	0.011
Serum TG	0.858	−0.353	−0.139
HDLC	−0.516	−0.578	0.402
LDLC	0.050	−0.094	−0.927
AST	0.922	−0.144	−0.026
Fecal TBA	−0.154	0.908	−0.008
Fecal TC	−0.171	0.842	−0.103
cAMP	0.040	−0.171	0.809
T	−0.422	−0.207	0.502

### 3.5. Histopathological analysis of liver

**Figure 5:**
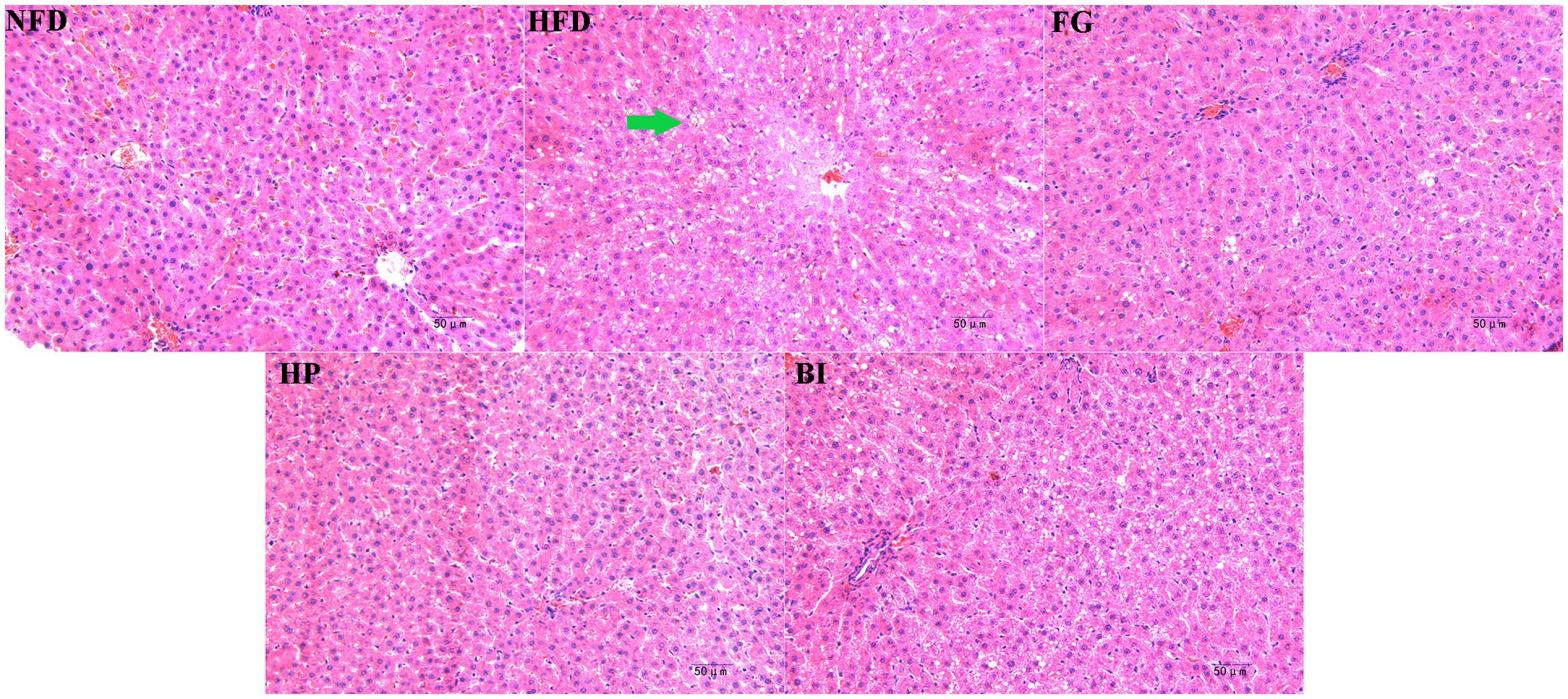
Histopathological analysis of liver tissue of rats in different groups at 200× magnification. The lipid droplet vacuoles in the hepatocytes has been marked by green arrows.

The histological morphology of the liver section in each group is shown in (Figure 5). In the NFD group, the liver cells of mice were arranged in an orderly manner and had normal size, the morphology of the liver cells was clear, and the cell membrane was complete, with sinuses opening at the central vein. On the contrary, the HFD group showed a large number of lipid droplet vacuoles in the hepatocytes, with a large intercellular space and disordered arrangement. After treatment with FG and HP, the morphology of the liver cells was significantly improved, the amount of lipid droplet vacuoles in the liver tissues was significantly reduced, the intercellular space became narrower, and the structure of the liver cells tended to be normal. The morphology of the liver tissue treatment with HP showed a similar state as that of the NFD group. Furthermore, the treated with BI had no effect on the liver in HFD-rats.

### 3.6. Intestinal bacteria analysis

#### 3.6.1. Effects of FG, HP and BI on intestinal bacteria

**Figure 6:**
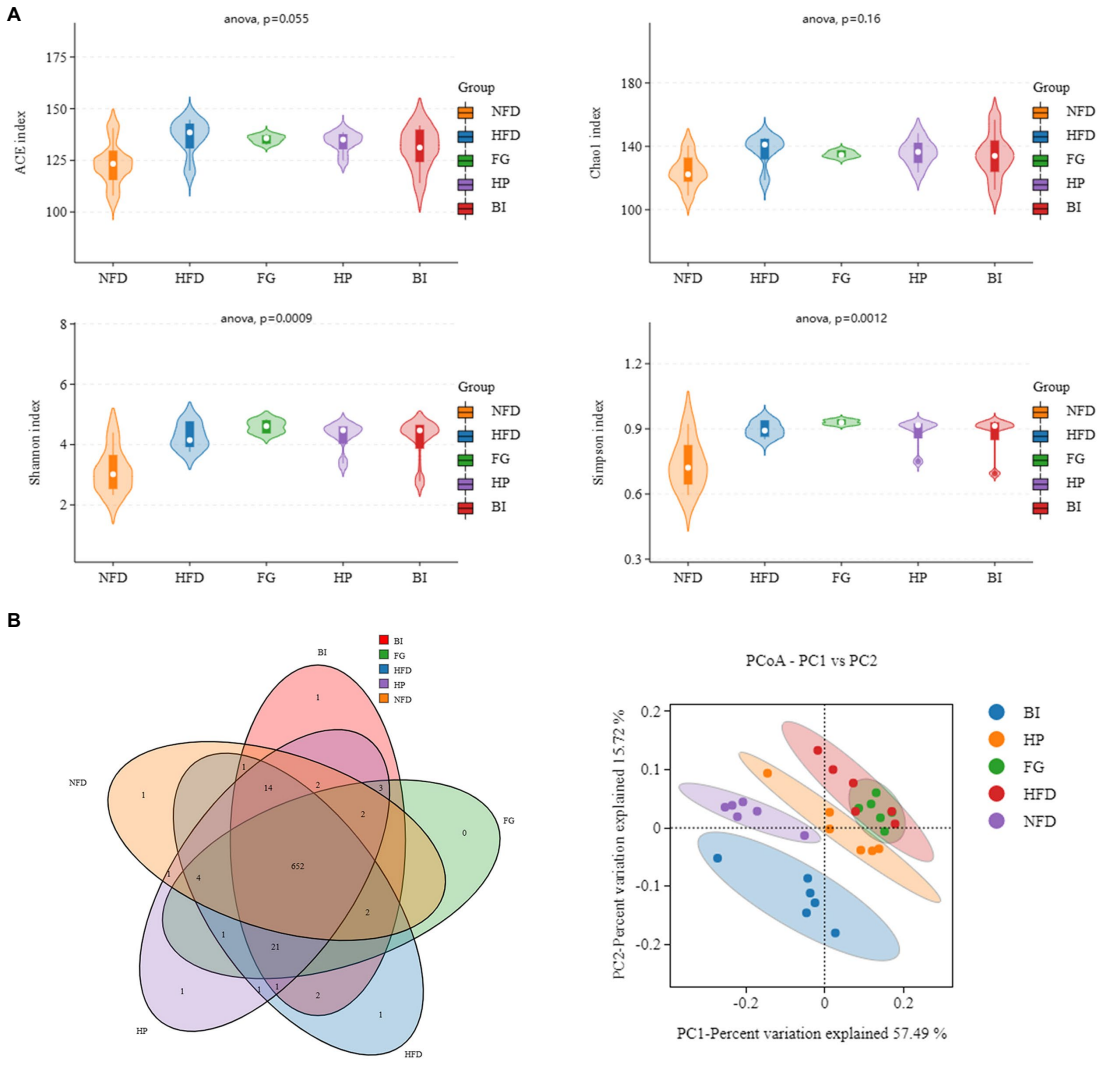
Diversity analysis of intestinal microflora in rat. **(A)** Alpha diversity violin (ACE, Chao1, Shannon, Simpson indice). **(B)** Venn diagram (NFD vs. HFD vs. FG vs. HP vs. BI) and Unweighted UniFrac PCoA.

Shannon and Simpson indexes were used to evaluate the bacterial diversity of gut microbiota, Chao 1 and abundance coverage-based estimator (ACE) indexes were used to evaluate the bacterial richness of gut microbiota. The abundance Shannon, Simpson, ACE, and Chao 1 indexes (Figure 6A) in the HFD were larger than those in the NFD groups (*p* < 0.05), indicating a higher microbial richness and diversity in the HFD group. However, compared with the three other groups, the effect on the four indexes were not significantly different. Meanwhile, a Venn diagram (Figure 6B) was used to better characterize the shared richness among the five groups. Principal coordinate analysis (PCoA) based on the weighted UniFrac distance was used to visualize the differences in the structure of gut microbiota. It was found that NFD and HFD groups could be clearly distinguished on the basis of the results of PCoA (Figure 6B). Compared with the HFD group, significant separation was also observed after HP and BI treatment, suggesting that HP and BI intervention could change the structure of gut microbiota after HFD treatment.

**Figure 7:**
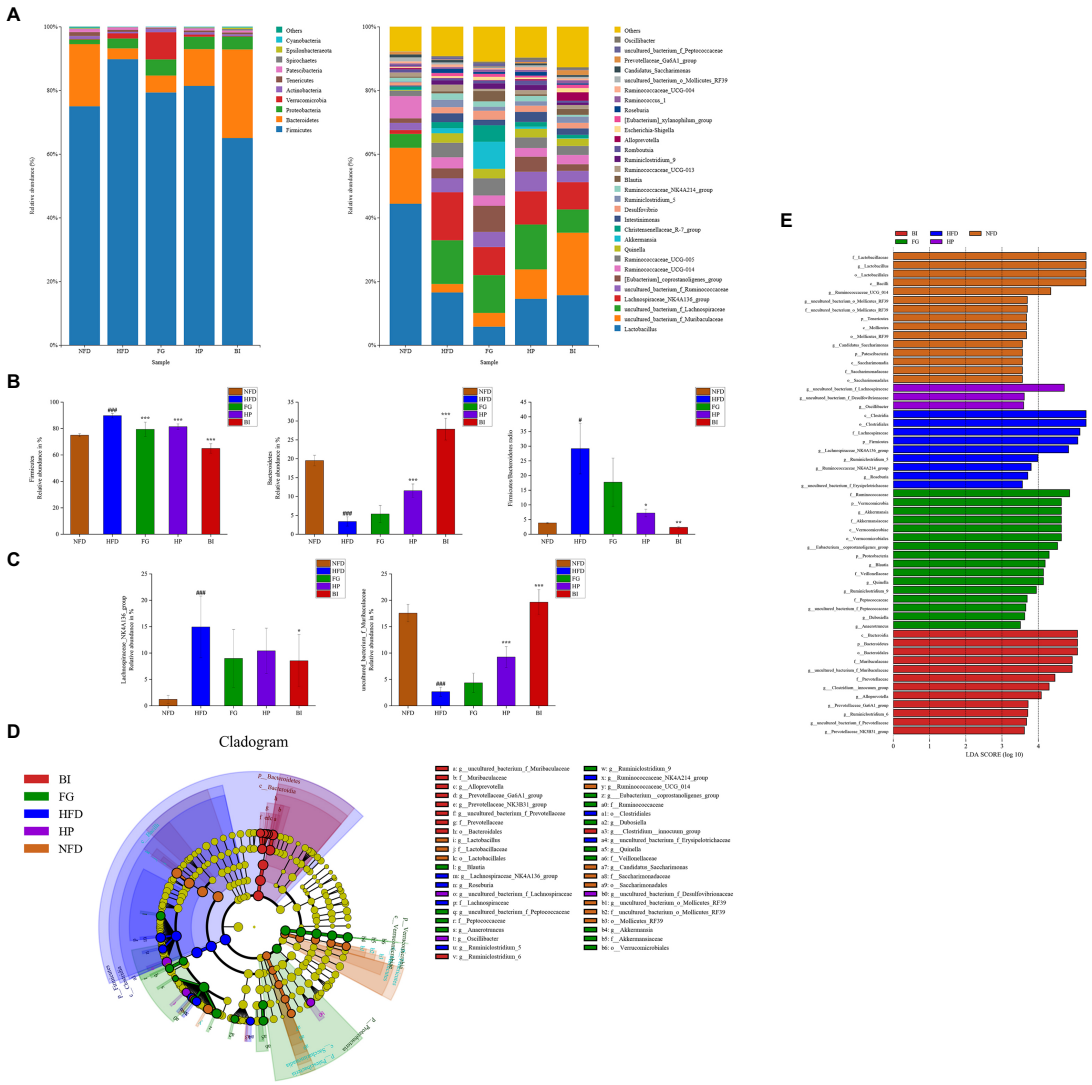
Effect of FG, HP and BI on the microbiota composition in HFD-fed rats. **(A)** Composition of intestinal microbiota at the phylum and genus level. **(B)** The abundance of Firmicutes, Bacteroidetes and the ratio of Firmicutes and Bacteroidetes in each group. **(C)** The abundance of Lachnospiraceae_ NK4A136_group, uncultured_bacterium_f_Muribaculaceae in each group. **(D)** LEfSe comparison of intestinal microbiota among five experimental groups. **(E)** Linear discriminant analysis (LDA) of five experimental groups. Significantly different from NFD group, #*p* < 0.05, ###*p* < 0.001. Significantly different from HFD group, *p < 0.05, **p < 0.01, ***p < 0.001.

As shown in (Figure 7A), at the phylum level, HFD significantly increased the population of Firmicutes and decreased Bacteroidetes. FG, HP, and BI treatment adjusted these imbalances, to be exact, decreased Firmicutes. And both HP and BI increased Bacteroidetes (Figure 7B). Furthermore, the ratio of Firmicutes and Bacteroidetes (F/B) was increased in HFD-fed rats compared with that of NFD-fed rats (3.854 vs. 29.176). The F/B ratio decreased to 7.239 and 2.361 after HP and BI supplementation, respectively, while FG had no effect on adjusting the ratio of F/B (Figure 7B). This suggested that HP and BI restored the intestinal bacteria balance to an extent.

The abundance of intestinal bacteria at the genus level of the five groups (NFD, HFD, FG, HP, and BI) are shown in (Figure 7A). The relative abundances of the top-abundant genus were significantly changed by HFD, while FG, HP, and BI restored the abundance of these bacteria to a certain extent. The difference at the genus level among the five groups (NFD, HFD, FG, HP, and BI) was analyzed by the Metastats test and shown in (Figure 7C). Fecal samples of the HFD group showed higher abundance of Lachnospiraceae_NK4A136_group (*p* < 0.001), but lower amount of uncultured_bacterium_f_Muribaculaceae (*p* < 0.001), as compared with the NFD group, which indicated intestinal microbial dysbiosis occurred in HFD-rats. And oral administration of HP and BI significantly increased the relative abundance of uncultured_bacterium_f_Muribaculaceae (*p* < 0.001). The level of was Lachnospiraceae_NK4A136_group significantly reduced by BI (*p* < 0.05).

The LEfSe analysis of the five groups (NFD, HFD, FG, HP, and BI) are shown in (Figure 7D). At the genus level, Lactobacillus, Ruminococcaceae_UCG_014, uncultured_bacterium_o_Mollicutes_RF39, and Candidatus_Saccharimonas were enriched in the NFD group. Lachnospiraceae_NK4A136_group, Ruminiclostridium_5, Ruminococcaceae_NK4A214_group, and Roseburia were enriched in the HFD group. Akkermansia, [Eubacterium]_coprostanoligenes_group, Blautia, Quinella, Ruminiclostridium_9, and uncultured_bacterium_f_Peptococcaceae were enriched in the FG group. And uncultured_bacterium_f_Lachnospiraceae, Oscillibacter were enriched in the HP group, whereas uncultured_bacterium_f_Muribaculaceae, Alloprevotella, and Prevotellaceae_Ga6A1_group were enriched in the BI group. The results of LEfSe analysis were consistent with linear discriminant analysis (LDA) as shown in (Figure 7E).

#### 3.6.2. Correlation analysis between dyslipidemia related indices and intestinal microbiota

**Figure 8:**
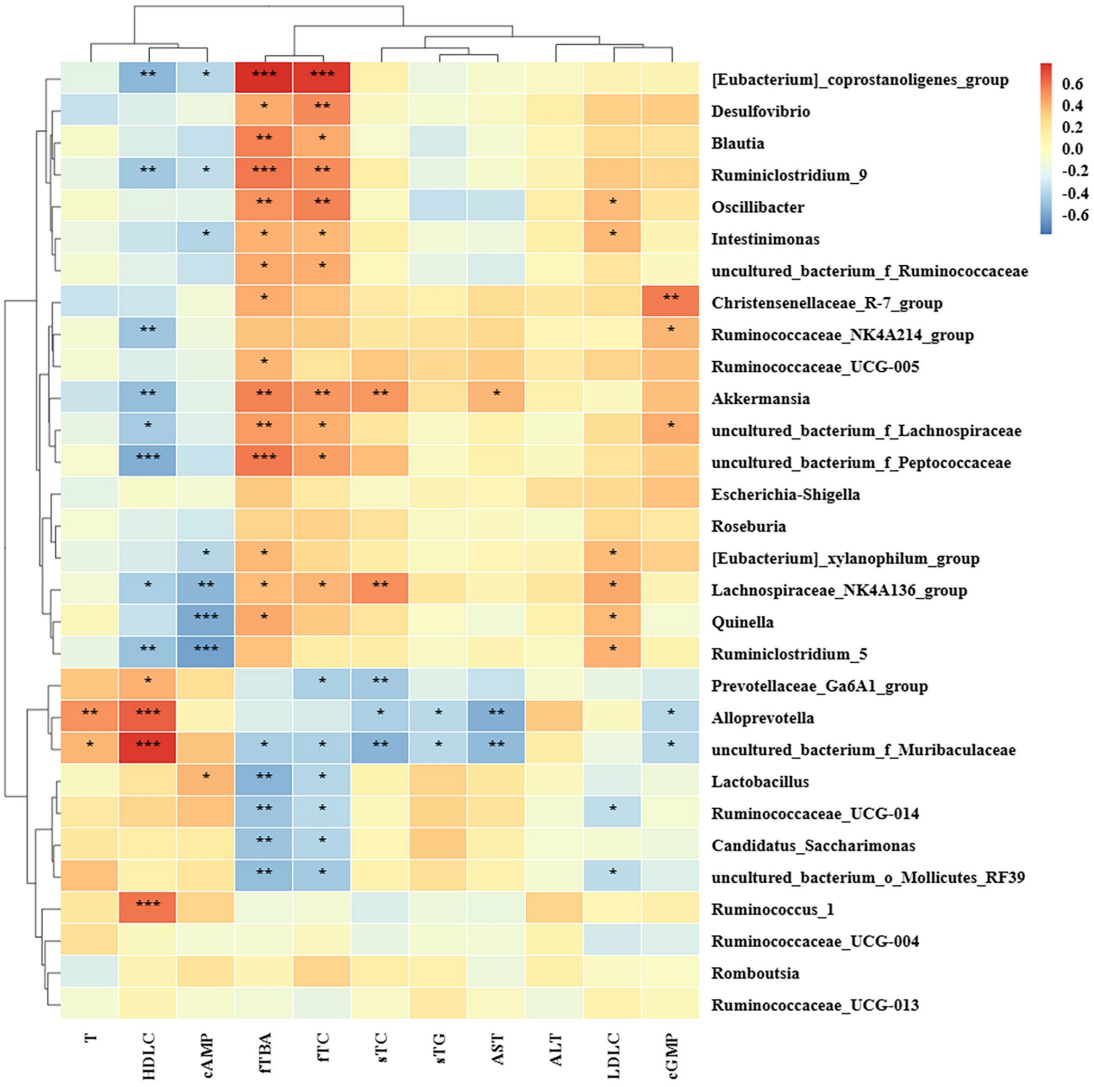
Correlation analysis between biochemical indiex and intestinal microbiota based on the Spearman’s analysis. sTC and sTG standed for serum TC and serum TG, respectively. fTC and fTBA standed for fecal TC and fecal TBA, respectively.

The correlation between biochemical parameters and rat intestinal microbiota at the genus level was studied based on Spearman’s analysis. As shown in (Figure 8), Lachnospiraceae_NK4A136_group was positively correlated with the levels of serum TC, LDL-C, fecal TC, TBA, and negatively correlated with the levels of serum HDL-C, cAMP, which significantly increased in the HFD group. In addition, the relative abundance of [Eubacterium]_coprostanoligenes_group, Desulfovibrio, Blautia, Ruminiclostridium_9, Oscillibacter, Intestinimonas, uncultured_bacterium_f_Ruminococcaceae, Akkermansia, uncultured_bacterium_f_Lachnospiraceae, uncultured_bacterium_f_Peptococcaceae, [Eubacterium]_xylanophilum_group, Quinella, Ruminococcaceae_UCG-005, and Christensenellaceae_R-7_group, showed positive correlation with fecal TC or TBA, while uncultured_bacterium_f_Muribaculaceae, Lactobacillus, Ruminococcaceae_UCG_014, Candidatus_Saccharimonas, and uncultured_bacterium_o_Mollicutes_RF39 showed negative correlation. Furthermore, Prevotellaceae_Ga6A1_group, Alloprevotella, and uncultured_bacterium_f_Muribaculaceae showed the negative correlation with serum TC or TG. Ruminococcus_1, uncultured_bacterium_f_Muribaculaceae, Alloprevotella, and Prevotellaceae_Ga6A1_group were positively correlated with serum HDL-C. Uncultured_bacterium_f_Peptococcaceae, [Eubacterium]_coprostanoligenes_group, uncultured_bacterium_f_Lachnospiraceae, Ruminiclostridium_9, Akkermansia, Ruminiclostridium_5, Ruminococcaceae_NK4A214_group, and Lachnospiraceae_NK4A136_group were negatively correlated with serum HDL-C. Oscillibacter, Quinella, Ruminiclostridium_5, Intestinimonas, Lachnospiraceae_NK4A136_group, and [Eubacterium]_xylanophilum_group were positively correlated with serum LDL-C, while Ruminococcaceae_UCG-014 and uncultured_bacterium_o_Mollicutes_RF39 were negatively correlated with it. As to liver injury index, Alloprevotella, uncultured_bacterium_f_Muribaculaceae were negatively correlated with AST, and Akkermansia was positively correlated with it. Ruminiclostridium_5, Quinella, Lachnospiraceae_NK4A136_group, Intestinimonas, [Eubacterium]_coprostanoligenes_group, [Eubacterium]_xylanophilum_group, and Ruminiclostridium_9 were negatively correlated with cAMP, and Lactobacillus was positively correlated with it. Alloprevotella and uncultured_bacterium_f_Muribaculaceae were positively correlated with T. Therefore, our results suggested that the genus was important in the regulation of lipid metabolism disorders.

## 4. Discussion

Hyperlipidemia is mainly manifested as increased serum TC, TG, LDL-C levels, decreased HDL-C levels, and usually accompanied by obesity, NAFLD, and other risks (28). The results of this study suggested that feeding FG, HP, and BI can treat HFD-induced hyperlipidemia by improving lipid metabolism, increasing beneficial intestinal bacteria, and modulating SCFAs levels, respectively. In this study, HFD resulted in rats with significantly increase in body weight, serum TC, TG, and LDL-C levels, and a significant decrease in HDL-C level. Although oral FG, HP and BI were no effect on the body weight of HFD rats, they significantly decreased the levels of TC and TG in the serum of the rats. Furthermore, FG significantly decreased the serum LDL-C level in rats. Notably, HP and BI significantly increased the serum HDL-C level. TC/HDL-C was used as an index to evaluate the risk of cardiovascular disease and atherosclerosis (29). FG, HP, and BI significantly restored HFD-induced HDL-C/TC changes indicating they could reduce the risk of cardiovascular disease.

Polysaccharides could affect the diffusion of cholesterol micelles due to changing the viscosity of the intestinal lumen, thereby reducing the bioavailability of cholesterol (18). The binding capacity between bile acids and polysaccharides has been ascribed to hydrophobic interactions, electrostatic interactions, hydrodynamic restriction, and entrapment of bile acids by polysaccharides (30). Polysaccharides inhibited the reabsorption of bile acids by the intestinal cells, reduced the enterohepatic circulation of bile acids, and promoted the conversion of cholesterol in the liver or blood into bile acids through binding with bile acids, thereby exerting the cholesterol-lowering effect (31, 32). Galactomannan sourced from fenugreek increased the viscosity of the small intestine and thus inhibited the absorption of cholesterol and bile salt (19, 33, 34). FG had higher substitution degree of galactose and linear structure, which endowed it with a stronger bile acid binding capacity (8, 35, 36). The formation of complexes of pectin and bile salts was related to its structure. The higher the degree of esterification, the more hydrophobic groups, and the stronger the binding ability to bile salts (37). But in the effect of cholesterol-lowering, the apparent viscosity rather than the degree of esterification of pectin was the critical factor (18). HP played a cholesterol-lowering effect by inhibiting the absorption of cholesterol and lipid and promoting the excretion of bile acids (13). Beyond its function as a BA sequestrant, pectin played a role in promoting bile acid synthesis and reducing bile acid taken up through increasing hepatic Cyp7a1 expression and decreasing ileal FXR activity (38). The results of this study were consisted of the above evidence, namely, the effects of lipid-lowering by FG and HP were realized by promoting the excretion of cholesterol. Inulin-type fructans with lower molecular weight and viscosity might not have the ability to bind to bile acids (39–42). Although organic acids fermented in the intestinal from inulin-type fructans have been shown to lower the pH in the intestinal and decrease the solubility of bile acids to increase their excretion from the feces, (43) BI had no significant effect on the excretion of TC and TBA in feces in our study. The cholesterol-lowering effect of inulin was attributed to the effect of promoting the production of liver unconjugated bile acids, but it could neither increase hepatobiliary cholesterol excretion nor decrease intestinal cholesterol uptake (44). This explained that BI decreased the level of cholesterol in the serum but did not increase the excretion of cholesterol.

Hyperlipidemia was often accompanied by lipid accumulation in the liver, which was induced accumulation and enlargement of hepatocytes, fatty infiltration, liver damage, and dysfunction, leading to an elevated liver index (45). Most studies have shown that HFD led to increased liver index, such as AST and ALT in mice or rats (46, 47). The ratio of AST/ALT was an indicator to measure the risk of alcoholic liver disease and gestational diabetes mellitus (48, 49).

Early research suggested that hawthorn pectin pentaoligosaccharide (HPPS) reduced liver damage caused by hyperlipidemia by boosting the liver’s antioxidant system (50). Inulin also has antioxidant properties that prevent CCl_4_-induced liver damage (51). In this study, FG and HP significantly restored the increase in AST levels and attenuated vacuolar lesions of hepatocytes caused by HFD feeding indicating that they could improve liver injury caused by HFD. In addition, they significantly decreased the ratio of AST/ALT in hyperlipidemia rats. Short-term inulin feeding could induce liver cholestasis and mild damage in HCD-mice (44). This might explain the abnormal decrease in the liver index and the similar state of vacuolar lesions of hepatocytes in liver histopathological sections of BI group and those of the HFD group. It was also reported that short-term inulin feeding had no effect on the level of ALT (44), which was consistent with our data. Since our results also showed that BI decreased the ratio of AST/ALT, which indicated a positive effect on liver injury, the effect of BI on HFD-induced liver injury needs to be confirmed by further studies. Although it was reported in the literature that the addition of 15% galactomannan in the daily diet aggravated liver damage caused by HCD-mice (52), the liver injury of FG was not found in our study, which might be due to the different administration dose and animal models (19). Therefore, the effects of FG on HFD-induced liver injury also needed further confirmation.

HFD led to pathological changes such as degeneration and atrophy of seminiferous tubules, and decreased spermatogenic cells in testicles (53, 54). Additionally, HCD decreased serum testosterone and estradiol levels in rats (55), and testosterone levels were negatively correlated with the risk of atherosclerosis (56). In this study, HFD feeding significantly decreased testicle index and testosterone levels in rats, which confirmed that HFD feeding had an effect on reproductive function in rats. FG significantly up-regulated the testis index in rats, and BI increased the level of testosterone in HFD rats. This suggested that FG and BI ameliorated HFD-induced reproductive damages.

In the process of fat mobilization, cAMP in adipocytes can activate lipolytic enzymes such as hormone-sensitive lipase (HSL) and adipocyte-specific triglyceride lipase (ATGL) by activating protein kinase A (PKA), and promote lipolysis (57). And cGMP enhanced brown/beige adipocyte-mediated thermogenesis as a targeted anti-obesity drug (58). In conclusion, cAMP and cGMP played important roles in the process of fat utilization and breakdown. The ratio of cAMP/cGMP significantly decreased in diabetic rats (59, 60). In this study, the ratio of cAMP/cGMP in HFD-fed rats significantly decreased, while HP and BI could significantly increase the ratio of cAMP/cGMP in rats, which indicated that HP and BI could decrease the diabetes risk induced by HFD.

Based on factor analysis, the differences in the effect of each group on hyperlipidemia were compared. In reducing blood lipid levels and improving hyperlipidemia (factor 1), HP and BI were stronger than FG. In regulating cholesterol excretion (factor 2), FG and HP played a significant role, and the effect of FG was stronger than that of HP. In addition, all three improved the impact on cholesterol transport and fat mobilization induced by HFD.

SCFAs were mainly produced by the fermentation of dietary fiber by intestinal microorganisms in the intestinal tract, composed of acetic acid, propionic acid and butyric acid, and they had the effect of regulating lipid metabolism (61). Acetic acid improved the absorption of glucose by skeletal muscle, reduced fasting and postprandial blood sugar, and played a role in the treatment of diabetes (62). In addition, acetic acid could also cross the blood–brain barrier to reduce appetite (63). Propionic acid not only inhibited hepatic lipid synthesis, and lowered cholesterol levels, but also mediated the release of intestinal hormones to improve insulin resistance (62, 64). Butyric acid was 70% of the energy source of intestinal epithelial cells, which affected the absorption and metabolism of intestinal cells and slowed down the transportation of intestinal fat (65, 66).

Early studies showed that HFD disrupted the balance of intestinal microbiota in rats, significantly decreasing the levels of total SCFAs and acetic acid in the intestinal (67). This was consistent with the results obtained in this study. In addition, HFD also resulted in decreased intestinal propionic acid and butyric acid levels in rats in this study. HP could significantly increase the level of total SCFAs in rats, restoring the total SCFAs concentration close to the normal value. Galacturonic acid, the skeleton component of HP, could be fermented to produce acetic acid and butyric acid by the intestinal microbiota (68). In addition, Ara in the side chain might promote the availability of HP in the fermentation to restore the concentration of total SCFAs in the caecum of HFD rats (69). In this study, the acetic acid level was slightly lower, while the levels of other SCFAs were not changed significantly in the FG group compared to the HFD group. This might be due to the higher Mw of FG, which was hard to be utilized by the intestinal microbiota (14, 70). Although BI was fermented by the intestinal microbiota to produce SCFAs such as propionic acid and butyric acid, the levels of most SCFAs in the BI group tended to decrease in this study, which was consistent with the results of Kiewiet’s study (16, 71). This result was attributed to the role of colonocytes in the absorption of SCFAs. However, in this study, caecal contents were used to measure the levels of SCFAs, and this possibility was ruled out. So the results might be due to the lower doses of BI applied in this study (16), and thus changes in SCFAs could not be detected.

Studies have shown that lipid metabolism disorders led to dysbiosis of the intestinal microbiota, and maintaining a stable intestinal microbial ecological environment regulated cholesterol metabolism in the liver, promoted lipid oxidation in muscle and energy storage in adipose tissue, and maintained the integrity of the intestinal barrier (72). At the phylum level, Firmicutes and Bacteroidetes are the dominant phylum of the intestinal microbiota. Firmicutes, the most abundance of gut microbiota, are responsible for energy resorption and obesity (73). Bacteroidetes primarily reside in the distal intestinal tract and contain a variety of polysaccharides and glycosidases and participate in the fermentation of indigestible polysaccharides, such as dietary fibers such as cellulose, hemicellulose, and β-glucan to produce SCFAs. They are the main carbohydrate-degrading bacteria in the gut (74). For example, Bacteroidetes was generally equipped with several enzymes able to degrade pectin, which could made good use of pectin components (75). During the degradation and utilization of polysaccharide fractions by the intestinal microbiota, the lower-molecular-weight polysaccharides were easier to be fermented (76). The fermentation rate of galactomannan by intestinal microflora was far lower than that of pectin, which might lead to the excretion of galactomannan in animal models with feces before being utilized by intestinal microflora (18). In contrast, FG with high molecular weight structure was more difficult to be fermented by Bacteroidetes, resulting in no significant change in the abundance of Bacteroidetes in the FG group. The ratio of Firmicutes to Bacteroidetes (F/B) increased in obesity, fatty liver, diabetes, hyperlipidemia, and other diseases (77–80). Consistent with most studies, HFD increased the F/B value in this study. HP and BI significantly decreased the F/B value indicating that HP and BI restored the balance of intestinal microbiota at the phylum level.

Lachnospiraceae_NK4A136_group, a distinguishing feature of gut dysbiosis, had higher levels in rats or mice with diseases such as type 2 diabetes, colitis, and NAFLD (81–84). Thus to decrease the relative abundance of Lachnospiraceae_NK4A136_group might be the key to slowing the development of these diseases (81). The level of Lachnospiracea_NK4A136_group, which was strongly related to hyperlipidemia indicators, improved high-fat diet-induced glucose and lipid metabolic disorders (85). However, other studies have shown that Lachnospiraceae_NK4A136_group as a kind of butyrate-producing bacteria, could maintain the integrity of the intestinal barrier, inhibit inflammation, and prevent obesity (86). Therefore, the role of the Lachnospiraceae_NK4A136_group in lipid-lowering was still controversial. According to the results of this study, Lachnospiraceae_NK4A136_group was positively correlated with the serum levels of TC and LDL-C and fecal levels of TC and TBA, negatively correlated with the level of serum HDL-C and cAMP, indicating that Lachnospiraceae_NK4A136_group as a harmful genus played a role in a disturbance the transportation of cholesterol in HFD-rats. BI significantly restored HFD-induced increase levels of them and improved cholesterol transportation. The uncultured_bacterium_f_Muribaculaceae was negatively correlated with blood lipid levels and associated with carbohydrate degradation, glycogen synthesis, and cellulose metabolism (87, 88). The correlation analysis in this study showed uncultured_bacterium_f_Muribaculaceae was positively correlated with levels of HDL-C, indicating it might relate to cholesterol transport. Furthermore, although uncultured_bacterium_f_Muribaculaceae showed the negative correlation with the levels of fecal TC and TBA, it was not the main genus for regulating the excretion of cholesterol. HP and BI significantly restored the decrease of the uncultured_bacterium_f_Muribaculaceae level.

Akkermansia is a member of the Verrucomicrobia phylum that is conducive to blood glucose stability. It can degrade mucin and reverse increases in fat mass caused by HFD, intra-metabolic toxemia, and insulin resistance, but it also can improve obesity through lipolysis, participation in the synthesis of secondary bile acids, and promotion bile acid metabolism (89–93). As a widely accepted beneficial genus, Blautia, Ruminiclostridium_9 were associated with anti-obesity effects (94, 95). In addition, [Eubacterium]_coprostanoligenes_group improves dyslipidemia induced by HFD through sphingosine supplementation (96). In addition, [Eubacterium]_coprostanoligenes_group regulated host lipid homeostasis partly by converting cholesterol to coprostanol to improve its excretion (96, 97). In the early study, fenugreek played a role in treating hyperlipidemia and improving insulin resistance by increasing the level of Akkermansia. In addition, the galactomannan from fenugreek also could promoted the growth of some beneficial microbes (98, 99). In this study Akkermansia, Blautia, Ruminiclostridium_9, and Quinella, etc. were enriched in the FG group. The results of the correlation analysis showed that the abundance of Akkermansia, Blautia, Ruminiclostridium_9, Quinella, [Eubacterium]_coprostanoligenes_group, and uncultured_bacterium_f_Peptococcaceae were positively correlated with the levels of fecal TC, TBA. This suggested that the effect of the promotion excretion of cholesterol in HFD-rats by FG was related to its effect on increasing beneficial genus in the intestinal.

Early studies have shown that lemon pectin could increase the level of Lachnospiraceae family in the human gut, but pectin with different esterification degrees and molecular weight had different effects on the intestinal flora (100). Uncultured_bacterium_f_Lachnospiraceae exhibited potential reduces the activity of fat deposits and metabolic disorders. Oscillibacter can ferment complex plant carbohydrates, and an increase in the abundance of Oscillospiraceae was significantly associated with the production of butyric acid (101). The above two genus were enriched in the HP group. The results of the correlation analysis showed that both of uncultured_bacterium_f_Lachnospiraceae and Oscillospiraceae were positively correlated with the levels of fecal TC, TBA.

In addition, Alloprevotella and Prevotellaceae_Ga6A1_group are SCFA-producing bacteria. Alloprevotella is a genus that ferments carbohydrates, which are negatively correlated with various diseases, such as obesity, diabetes, and cardiovascular diseases (102). In this study, uncultured_bacterium_f_Muribaculaceae, Alloprevotella, and Prevotellaceae_Ga6A1_group, which enriched in the BI group were negatively correlated with the levels of serum TC, TG, AST, and positively correlated with the levels of serum HDL-C. In addition, Prevotellaceae_NK3B31_group, uncultured_bacterium_f_Prevotellaceae, which are from Prevotellaceae also enriched in the BI group. The higher abundance of Prevotellaceae not only protects intestinal epithelial cells against oxidative stress, thereby, alleviating inflammatory symptoms in patients with inflammatory bowel diseases, but also beneficial for preventing obesity (103, 104). Consistent with this study, *Jerusalem artichoke* inulin also enriched several genera of the Prevotelaceae family, and improved lipid metabolism in type 2 diabetes mice (105). This suggested that Prevotellaceae might be the key family for BI to exert its effect on lipid-lowering.

The results showed that FG, HP and BI had good lipid-lowering activity. Among them, FG and HP with relatively high molecular weight played the lipid-lowering roles by increasing the excretion of cholesterol and bile acid. In the role of regulating the intestinal microenvironment, HP could be fermented and utilized by intestinal flora to produce SCFAs. HP and BI could also restore intestinal flora disorder caused by HFD. Although FG with large molecular weight was difficult to be used by most intestinal flora, it might still be specifically used by the several kinds of intestinal bacteria. In this study, FG enriched some beneficial bacteria. This study analyzed the differences in some mechanisms of FG, HP, and BI in the treatment of hyperlipidemia, but did not include all lipid-lowering pathways. Therefore, the effect of different polysaccharides in the treatment of hyperlipidemia needs further study.

## Data availability statement

The datasets presented in this study can be found in online repositories. The names of the repository/repositories and accession number(s) can be found at: https://www.ncbi.nlm.nih.gov/, PRJNA925574.

## Ethics statement

The animal study was reviewed and approved by the Liaoning University of Traditional Chinese Medicine Research Ethics Committee.

## Author contributions

YW: data curation and writing – original draft preparation. YZ: visualization and methodology. YL, BZ, and JL: investigation. GS: validation. QC: conceptualization. YQ: writing – reviewing and editing and supervision. All authors contributed to the article and approved the submitted version.

## Funding

This work was supported by the National Natural Science Foundation of China (82104393), Liaoning Revitalization Talents Program (XLYC2002004), and the Foundation from the Department of Education of Liaoning Province (201949).

## Conflict of interest

The authors declare that the research was conducted in the absence of any commercial or financial relationships that could be construed as a potential conflict of interest.

## Publisher’s note

All claims expressed in this article are solely those of the authors and do not necessarily represent those of their affiliated organizations, or those of the publisher, the editors and the reviewers. Any product that may be evaluated in this article, or claim that may be made by its manufacturer, is not guaranteed or endorsed by the publisher.

## Footnotes

^1^ www.biocloud.net

## References

[1] Yu W Shi R Li J Lan Y Li Q Hu S. Need for hyperlipidemia management policy reform in China: learning from the global experience. Curr Med Res Opin. (2018) 34:197–207. doi: 10.1080/03007995.2017.1354833, PMID: 28696793

[2] Kajinami K Tsukamoto K Koba S Inoue I Yamakawa M Suzuki S. Statin intolerance clinical guide 2018. J Atheroscler Thromb. (2020) 27:375–96. doi: 10.5551/jat.50948, PMID: 31588101 PMC7192817

[3] Zhang L Wang X Zhang X. Modulation of intestinal flora by dietary polysaccharides: a novel approach for the treatment and prevention of metabolic disorders. Foods. (2022) 11:2961. doi: 10.3390/foods11192961, PMID: 36230037 PMC9562892

[4] Nie Y Luo F. Dietary fiber: an opportunity for a global control of hyperlipidemia. Oxidative Med Cell Longev. (2021) 2021:5542342. doi: 10.1155/2021/5542342, PMID: 33897940 PMC8052145

[5] Jian H Lin X Zhang W Zhang W Sun D Jiang J. Characterization of fractional precipitation behavior of galactomannan gums with ethanol and isopropanol. Food Hydrocoll. (2014) 40:115–21. doi: 10.1016/j.foodhyd.2014.02.012

[6] Guo Q Du J Jiang Y Goff HD Cui SW. Pectic polysaccharides from hawthorn: physicochemical and partial structural characterization. Food Hydrocoll. (2019) 90:146–53. doi: 10.1016/j.foodhyd.2018.10.011

[7] Li L Qiu Z Dong H Ma C Qiao Y Zheng Z. Structural characterization and antioxidant activities of one neutral polysaccharide and three acid polysaccharides from the roots of *Arctium lappa* L.: a comparison. Int J Biol Macromol. (2021) 182:187–96. doi: 10.1016/j.ijbiomac.2021.03.177, PMID: 33836197

[8] Salarbashi D Bazeli J Fahmideh-Rad E. Fenugreek seed gum: biological properties, chemical modifications, and structural analysis - A review. Int J Biol Macromol. (2019) 138:386–93. doi: 10.1016/j.ijbiomac.2019.07.006, PMID: 31276725

[9] Rahnama F Mohammadzadeh Milani J Gohari AA. Improved quality attributes of brabari and lavash flat breads with wheat doughs incorporated with fenugreek seed (*Trigonella foenum graecum* L) Gum. J Food Process Preserv. (2017) 41:e12741. doi: 10.1111/jfpp.12741

[10] Li L Gao X Liu J Chitrakar B Wang B Wang Y. Hawthorn pectin: extraction, function and utilization. Curr Res Food Sci. (2021) 4:429–35. doi: 10.1016/j.crfs.2021.06.002, PMID: 34258587 PMC8253901

[11] Singla V Chakkaravarthi S. Applications of prebiotics in food industry: a review. Food Sci Technol Int. (2017) 23:649–67. doi: 10.1177/1082013217721769 28762780

[12] Srichamroen A Thomson AB Field CJ Basu TK. In vitro intestinal glucose uptake is inhibited by galactomannan from Canadian fenugreek seed (*Trigonella foenum graecum* L) in genetically lean and obese rats. Nutr Res. (2009) 29:49–54. doi: 10.1016/j.nutres.2008.11.002, PMID: 19185777

[13] Zhu RG Sun YD Li TP Chen G Peng X Duan WB. Comparative effects of hawthorn (*Crataegus pinnatifida Bunge*) pectin and pectin hydrolyzates on the cholesterol homeostasis of hamsters fed high-cholesterol diets. Chem Biol Interact. (2015) 238:42–7. doi: 10.1016/j.cbi.2015.06.006, PMID: 26070415

[14] Shtriker MG Hahn M Taieb E Nyska A Moallem U Tirosh O. Fenugreek galactomannan and citrus pectin improve several parameters associated with glucose metabolism and modulate gut microbiota in mice. Nutrition. (2018) 46:134–142.e3. doi: 10.1016/j.nut.2017.07.012, PMID: 28993009

[15] Liu T Cui T Gao Z. Recent advances in dietary fiber of hawthorn. Food Res Dev. (2020) 41:199–203.

[16] Watanabe A Sasaki H Miyakawa H Nakayama Y Lyu Y Shibata S. Effect of dose and timing of burdock (*Arctium lappa*) root intake on intestinal microbiota of mice. Microorganisms. (2020) 8:220. doi: 10.3390/microorganisms8020220, PMID: 32041173 PMC7074855

[17] Ke J An Y Cao B Lang J Wu N Zhao D. Orlistat-induced gut microbiota modification in obese mice. Evid Based Complement Alternat Med. (2020) 2020:1–9. doi: 10.1155/2020/9818349, PMID: 32328145 PMC7168719

[18] Silva IMV Machado F Moreno MJ Nunes C Coimbra MA Coreta-Gomes F. Polysaccharide structures and their hypocholesterolemic potential. Molecules. (2021) 26:4559. doi: 10.3390/molecules26154559, PMID: 34361718 PMC8348680

[19] Hamden K Jaouadi B Carreau S Bejar S Elfeki A. Inhibitory effect of fenugreek galactomannan on digestive enzymes related to diabetes, hyperlipidemia, and liver-kidney dysfunctions. Biotechnol Bioprocess Eng. (2010) 15:407–13. doi: 10.1007/s12257-009-3037-9

[20] Wang N Zhang C Qi Y Li T. Extraction of hawthorn pectin and its food chemical properties. Sci Technol Food Ind. (2007) 1:87–9. doi: 10.13386/j.issn1002-0306.2007.11.016

[21] Zhang A Shen Y Cen M Hong X Shao Q Chen Y. Polysaccharide and crocin contents, and antioxidant activity of saffron from different origins. Ind Crop Prod. (2019) 133:111–7. doi: 10.1016/j.indcrop.2019.03.009

[22] Bradford MM. A rapid and sensitive method for the quantitation of microgram quantities of protein utilizing the principle of protein-dye binding. Anal Biochem. (1976) 72:248–54. doi: 10.1016/0003-2697(76)90527-3, PMID: 942051

[23] Li L Guo WL Zhang W Xu JX Qian M Bai WD. *Grifola frondosa* polysaccharides ameliorate lipid metabolic disorders and gut microbiota dysbiosis in high-fat diet fed rats. Food Funct. (2019) 10:2560–72. doi: 10.1039/c9fo00075e, PMID: 30994668

[24] Chen K Luan X Liu Q Wang J Chang X Snijders AM. Drosophila histone demethylase KDM5 regulates social behavior through immune control and gut microbiota maintenance. Cell Host Microbe. (2019) 25:537–552.e8. doi: 10.1016/j.chom.2019.02.003, PMID: 30902578 PMC6749836

[25] Yang C Xu Z Deng Q Huang Q Wang X Huang F. Beneficial effects of flaxseed polysaccharides on metabolic syndrome via gut microbiota in high-fat diet fed mice. Food Res Int. (2020) 131:108994. doi: 10.1016/j.foodres.2020.108994, PMID: 32247451

[26] Sheng D Zhao S Gao L Zheng H Liu W Hou J. Babao Dan attenuates high-fat diet-induced non-alcoholic fatty liver disease via activation of AMPK signaling. Cell Biosci. (2019) 9:77. doi: 10.1186/s13578-019-0339-2, PMID: 31548878 PMC6751621

[27] Wang JB Zhao HP Zhao YL Jin C Liu DJ Kong WJ. Hepatotoxicity or hepatoprotection? Pattern recognition for the paradoxical effect of the Chinese herb *Rheum palmatum* L. in treating rat liver injury. PLoS One. (2011) 6:e24498. doi: 10.1371/journal.pone.0024498, PMID: 21915343 PMC3167848

[28] Hazer I Kabukcu HO Yagci M Erturk Z Yildirim GK Kirel B. The association of lipid metabolism and non-alcoholic fatty liver disease in children with obesity. Turk Pediatri Ars. (2020) 55:263–9. doi: 10.14744/TurkPediatriArs.2020.65148, PMID: 33061754 PMC7536461

[29] Calling S Johansson S-E Wolff M Sundquist J Sundquist K. Total cholesterol/HDL-C ratio versus non-HDL-C as predictors for ischemic heart disease: a 17-year follow-up study of women in southern Sweden. BMC Cardiovasc Disord. (2021) 21:163. doi: 10.1186/s12872-021-01971-1, PMID: 33820540 PMC8020530

[30] Torcello-Gomez A Fernandez Fraguas C Ridout MJ Woodward NC Wilde PJ Foster TJ. Effect of substituent pattern and molecular weight of cellulose ethers on interactions with different bile salts. Food Funct. (2015) 6:730–9. doi: 10.1039/c5fo00099h, PMID: 25679293

[31] Wu Q Wang Q Fu J Ren R. Polysaccharides derived from natural sources regulate triglyceride and cholesterol metabolism: a review of the mechanisms. Food Funct. (2019) 10:2330–9. doi: 10.1039/c8fo02375a, PMID: 31049523

[32] Kahlon TS Smith GE Shao Q. In vitro binding of bile acids by kidney bean (*Phaseolus vulgaris*), black gram (*Vigna mungo*), bengal gram (*Cicer arietinum*) and moth bean (*Phaseolus aconitifolins*). Food Chem. (2005) 90:241–6. doi: 10.1016/j.foodchem.2004.03.046

[33] Rideout TC Harding SV Jones PJ Fan MZ. Guar gum and similar soluble fibers in the regulation of cholesterol metabolism: current understandings and future research priorities. Vasc Health Risk Manage. (2008) 4:1023–33. doi: 10.2147/vhrm.s3512, PMID: 19183750 PMC2605338

[34] Mendis M Simsek S. Arabinoxylans and human health. Food Hydrocoll. (2014) 42:239–43. doi: 10.1016/j.foodhyd.2013.07.022

[35] Roshan I Nagori BP. Effect of different galactomannans on absorption of cholesterol in rabbits. J Nat Rem. (2006) 6:86.

[36] Yang Y Zhao M Lin L. Effects of extraction methods on structural characteristics and bile acid-binding capacities of *Moringa oleifera* leaf polysaccharide fractions. Int J Food Sci Technol. (2020) 55:1539–46. doi: 10.1111/ijfs.14430

[37] Mumtaz Hamdani A Ahmed WI. Guar and Locust bean gum: Composition, total phenolic content, antioxidant and antinutritional characterisation. Bioact Carbohydr Diet Fibre. (2017) 11:53–9. doi: 10.1016/j.bcdf.2017.07.004

[38] Ciocan D Spatz M Trainel N Hardonnière K Domenichini S Mercier-Nomé F. Modulation of the bile acid enterohepatic cycle by intestinal microbiota alleviates alcohol liver disease. Cells. (2022) 11:968. doi: 10.3390/cells11060968, PMID: 35326419 PMC8946080

[39] Wu DT Zhao YX Guo H Gan RY Peng LX Zhao G. Physicochemical and biological properties of polysaccharides from *Dictyophora indusiata* prepared by different extraction techniques. Polymers. (2021) 13:2357. doi: 10.3390/polym13142357, PMID: 34301113 PMC8309502

[40] Schmidt M Sciurba E Nikolay S Hüsken A Smit I. Relevance of β-Glucan molecular properties on its suitability as health promoting bread ingredient. Nutrients. (2022) 14:1570. doi: 10.3390/nu14081570, PMID: 35458132 PMC9032243

[41] Schneeman B. Fiber, inulin and oligofructose: Similarities and differences. J Nutr. (1999) 129:1424S–7S. doi: 10.1093/jn/129.7.1424S, PMID: 10395611

[42] Mistry RH Gu F Schols HA Verkade HJ Tietge UJF. Effect of the prebiotic fiber inulin on cholesterol metabolism in wildtype mice. Sci Rep. (2018) 8:13238. doi: 10.1038/s41598-018-31698-7, PMID: 30185894 PMC6125380

[43] Li L Li P Xu L. Assessing the effects of inulin-type fructan intake on body weight, blood glucose, and lipid profile: a systematic review and meta-analysis of randomized controlled trials. Food Sci Nutr. (2021) 9:4598–616. doi: 10.1002/fsn3.2403, PMID: 34401107 PMC8358370

[44] Pauly MJ Rohde JK John C Evangelakos I Koop AC Pertzborn P. Inulin supplementation disturbs hepatic cholesterol and bile acid metabolism independent from housing temperature. Nutrients. (2020) 12:3200. doi: 10.3390/nu12103200, PMID: 33092056 PMC7589137

[45] Huang Y Wang N Zhao H. In vivo activities of the structured lipids −1, 3-dioleic acid 2-palmitic acid triglyceride (OPO) in high-fat diet mice. Food Biosci. (2022) 47:101667. doi: 10.1016/j.fbio.2022.101667

[46] Wei F Liu Y Bi C Zhang B. *Nostoc sphaeroids* Kutz powder ameliorates diet-induced hyperlipidemia in C57BL/6j mice. Food Nutr Res. (2019) 63:3618–3628. doi: 10.29219/fnr.v63.3618, PMID: 31920470 PMC6939666

[47] Du H Li C Wang Z He Y Wang Y Zhou H. Effects of Danhong injection on dyslipidemia and cholesterol metabolism in high-fat diets fed rats. J Ethnopharmacol. (2021) 274:114058. doi: 10.1016/j.jep.2021.114058, PMID: 33766756

[48] Gurung R Purbe B Gyawali P Risal P. The ratio of aspartate aminotransferase to alanine aminotransferase (AST/ALT): the correlation of value with underlying severity of alcoholic liver disease. Kathmandu Univ Med J. (2013) 11:233–6. doi: 10.3126/kumj.v11i3.12511, PMID: 24442172

[49] Liu H Zha X Ding C Hu L Li M Yu Y. AST/ALT ratio and peripheral artery disease in a Chinese hypertensive population: a cross-sectional study. Angiology. (2021) 72:916–22. doi: 10.1177/00033197211004410, PMID: 33779311

[50] Li TP Zhu RG Dong YP Liu YH Li SH Chen G. Effects of pectin pentaoligosaccharide from Hawthorn (*Crataegus pinnatifida Bunge*. var. Major) on the activity and mRNA levels of enzymes involved in fatty acid oxidation in the liver of mice fed a high-fat diet. J Agric Food Chem. (2013) 61:7599–605. doi: 10.1021/jf400283w, PMID: 23855516

[51] Correa-Ferreira ML Verdan MH Dos Reis Livero FA Galuppo LF Telles JE Alves Stefanello ME. Inulin-type fructan and infusion of *Artemisia vulgaris* protect the liver against carbon tetrachloride-induced liver injury. Phytomedicine. (2017) 24:68–76. doi: 10.1016/j.phymed.2016.11.017, PMID: 28160864

[52] Shtriker MG Peri I Taieb E Nyska A Tirosh O Madar Z. Galactomannan more than pectin exacerbates liver injury in mice fed with high-fat, high-cholesterol diet. Mol Nutr Food Res. (2018) 62:e1800331. doi: 10.1002/mnfr.201800331, PMID: 30051965

[53] Arisha SM Sakr SA Abd-Elhaseeb FR. *Cinnamomum zeylanicum* alleviate testicular damage induced by high fat diet in albino rats; histological and ultrastructural studies. Heliyon. (2020) 6:e05584. doi: 10.1016/j.heliyon.2020.e05584, PMID: 33294709 PMC7695915

[54] Sozen E Demirel-Yalciner T Koroglu MK Elmas MA Ercan F Ozer NK. High cholesterol diet activates ER stress mediated apoptosis in testes tissue: role of alpha-tocopherol. IUBMB Life. (2022) 74:85–92. doi: 10.1002/iub.2535, PMID: 34350697

[55] Wannasiri S Chansakaow S Sireeratawong S. Effects of *Solanum torvum* fruit water extract on hyperlipidemia and sex hormones in high-fat fed male rats. Asian Pac J Trop Biomed. (2017) 7:401–5. doi: 10.1016/j.apjtb.2017.01.027

[56] Rezanezhad B Borgquist R Willenheimer R Elzanaty S. The association between serum testosterone and risk factors for atherosclerosis. Curr Urol. (2019) 13:101–6. doi: 10.1159/000499285, PMID: 31768177 PMC6873033

[57] Ravnskjaer K Madiraju A Montminy M. Role of the cAMP pathway in glucose and lipid metabolism. Handb Exp Pharmacol. (2016) 233:29–49. doi: 10.1007/164_2015_32 26721678

[58] Reverte-Salisa L Sanyal A Pfeifer A. Role of cAMP and cGMP signaling in brown fat. Handb Exp Pharmacol. (2019) 251:161–82. doi: 10.1007/164_2018_117, PMID: 29633180

[59] Zhao J Cai CK Xie M Liu JN Wang BZ. Investigation of the therapy targets of Yi-Qi-Yang-Yin-Hua-Tan-Qu-Yu recipe on type 2 diabetes by serum proteome labeled with iTRAQ. J Ethnopharmacol. (2018) 224:1–14. doi: 10.1016/j.jep.2018.03.027, PMID: 29654829

[60] Zhao J Liu J Wang B Yao Y Zhang G Liu B. Efficacy of integrative medicine in deficiency of both qi and yin in the rat model of type 2 diabetes. J Tradit Chin Med Sci. (2015) 2:258–63. doi: 10.1016/j.jtcms.2016.01.013

[61] Markowiak-Kopec P Slizewska K. The effect of probiotics on the production of short-chain fatty acids by human intestinal microbiome. Nutrients. (2020) 12:1107. doi: 10.3390/nu12041107 32316181 PMC7230973

[62] Onyszkiewicz M Jaworska K Ufnal M. Short chain fatty acids and methylamines produced by gut microbiota as mediators and markers in the circulatory system. Exp Biol Med. (2020) 245:166–75. doi: 10.1177/1535370219900898, PMID: 31948289 PMC7016413

[63] Frost G Sleeth ML Sahuri-Arisoylu M Lizarbe B Cerdan S Brody L. The short-chain fatty acid acetate reduces appetite via a central homeostatic mechanism. Nat Commun. (2014) 5:3611. doi: 10.1038/ncomms4611, PMID: 24781306 PMC4015327

[64] Chambers ES Viardot A Psichas A Morrison DJ Murphy KG Zac-Varghese SE. Effects of targeted delivery of propionate to the human colon on appetite regulation, body weight maintenance and adiposity in overweight adults. Gut. (2015) 64:1744–54. doi: 10.1136/gutjnl-2014-307913, PMID: 25500202 PMC4680171

[65] Serpa J Caiado F Carvalho T Torre C Goncalves LG Casalou C. Butyrate-rich colonic microenvironment is a relevant selection factor for metabolically adapted tumor cells. J Biol Chem. (2010) 285:39211–23. doi: 10.1074/jbc.M110.156026, PMID: 20926374 PMC2998102

[66] Marcil V Delvin E Garofalo C Levy E. Butyrate impairs lipid transport by inhibiting microsomal triglyceride transfer protein in Caco-2 cells. J Nutr. (2003) 133:2180–3. doi: 10.1093/jn/133.7.2180, PMID: 12840175

[67] Wang S Li Q Zang Y Zhao Y Liu N Wang Y. Apple Polysaccharide inhibits microbial dysbiosis and chronic inflammation and modulates gut permeability in HFD-fed rats. Int J Biol Macromol. (2017) 99:282–92. doi: 10.1016/j.ijbiomac.2017.02.074, PMID: 28238909

[68] Chen C Huang Q Fu X Liu RH. In vitro fermentation of mulberry fruit polysaccharides by human fecal inocula and impact on microbiota. Food Funct. (2016) 7:4637–43. doi: 10.1039/c6fo01248e, PMID: 27748781

[69] Wu D Chen S Ye X Ahmadi S Hu W Yu C. Protective effects of six different pectic polysaccharides on DSS-induced IBD in mice. Food Hydrocoll. (2022) 127:107209. doi: 10.1016/j.foodhyd.2021.107209

[70] Mao YH Xu YX Li YH Cao J Song FL Zhao D. Effects of konjac glucomannan with different molecular weights on gut microflora with antibiotic perturbance in in vitro fecal fermentation. Carbohydr Polym. (2021) 273:118546. doi: 10.1016/j.carbpol.2021.118546, PMID: 34560958

[71] Kiewiet MBG Elderman ME El Aidy S Burgerhof JGM Visser H Vaughan EE. Flexibility of gut microbiota in ageing individuals during dietary fiber long-chain inulin intake. Mol Nutr Food Res. (2021) 65:e2000390. doi: 10.1002/mnfr.202000390, PMID: 33369019 PMC8138623

[72] Jia X Xu W Zhang L Li X Wang R Wu S. Impact of gut microbiota and microbiota-related metabolites on hyperlipidemia. Front Cell Infect Microbiol. (2021) 11:634780. doi: 10.3389/fcimb.2021.634780, PMID: 34490132 PMC8417472

[73] Peng F Ren X Du B Niu K Yu Z Yang Y. Insoluble dietary fiber of pear fruit pomace (*Pyrus ussuriensis Maxim*) consumption ameliorates alterations of the obesity-related features and gut microbiota caused by high-fat diet. J Funct Foods. (2022) 99:105354. doi: 10.1016/j.jff.2022.105354

[74] Yang X Bao L Zhang Y Long J Li Y Wang H. Novel weight loss diet attenuates dietary-induced obesity in mice and might correlate with altered gut microbiota and metabolite profiles. Front Nutr. (2022) 9:2784. doi: 10.3389/fnut.2022.987955, PMID: 36438747 PMC9692001

[75] Pascale N Gu F Larsen N Jespersen L Respondek F. The potential of pectins to modulate the human gut microbiota evaluated by in vitro fermentation: a systematic review. Nutrients. (2022) 14:3629. doi: 10.3390/nu14173629, PMID: 36079886 PMC9460662

[76] Li Q Dou Z Chen C Jiang Y Yang B Fu X. Study on the effect of molecular weight on the gut microbiota fermentation properties of blackberry polysaccharides in vitro. J Agric Food Chem. (2022) 70:11245–57. doi: 10.1021/acs.jafc.2c03091, PMID: 36053142

[77] Di Pierro F. Gut microbiota parameters potentially useful in clinical perspective. Microorganisms. (2021) 9:2402. doi: 10.3390/microorganisms9112402, PMID: 34835527 PMC8623243

[78] Zhao L Qi Z Yi L Li J Cui Y Ur Rehman F. The interaction between gut microbiota and flavonoid extract from *Smilax glabra* Roxb. and its potent alleviation of fatty liver. Food Funct. (2021) 12:7836–50. doi: 10.1039/d1fo00727k, PMID: 34235516

[79] Zhao C Qu Q Yang F Li Z Yang P Han L. Monascus ruber fermented *Panax ginseng* ameliorates lipid metabolism disorders and modulate gut microbiota in rats fed a high-fat diet. J Ethnopharmacol. (2021) 278:114300. doi: 10.1016/j.jep.2021.114300, PMID: 34098018

[80] Xu J Lian F Zhao L Zhao Y Chen X Zhang X. Structural modulation of gut microbiota during alleviation of type 2 diabetes with a Chinese herbal formula. ISME J. (2015) 9:552–62. doi: 10.1038/ismej.2014.177, PMID: 25279787 PMC4331591

[81] Wang K Li B Fu R Jiang Z Wen X Ni Y. Bentong ginger oleoresin mitigates liver injury and modulates gut microbiota in mouse with nonalcoholic fatty liver disease induced by high-fat diet. J Food Sci. (2022) 87:1268–81. doi: 10.1111/1750-3841.16076, PMID: 35152443

[82] Cui HX Zhang LS Luo Y Yuan K Huang ZY Guo Y. A purified anthraquinone-glycoside preparation from rhubarb ameliorates type 2 diabetes mellitus by modulating the gut microbiota and reducing inflammation. Front Microbiol. (2019) 10:1423. doi: 10.3389/fmicb.2019.01423, PMID: 31293553 PMC6603233

[83] Yao Q Fan L Zheng N Blecker C Delcenserie V Li H. 2′-fucosyllactose ameliorates inflammatory bowel disease by modulating gut microbiota and promoting MUC2 expression. Front Nutr. (2022) 9:822020. doi: 10.3389/fnut.2022.822020, PMID: 35252301 PMC8892212

[84] Zheng J Yuan X Cheng G Jiao S Feng C Zhao X. Chitosan oligosaccharides improve the disturbance in glucose metabolism and reverse the dysbiosis of gut microbiota in diabetic mice. Carbohydr Polym. (2018) 190:77–86. doi: 10.1016/j.carbpol.2018.02.058, PMID: 29628262

[85] Du L Wang Q Ji S Sun Y Huang W Zhang Y. Metabolomic and microbial remodeling by Shanmei capsule improves hyperlipidemia in high fat food-induced mice. Front Cell Infect Microbiol. (2022) 12:449. doi: 10.3389/fcimb.2022.729940, PMID: 35573781 PMC9094705

[86] Wang Q He Y Li X Zhang T Liang M Wang G. Lactobacillus reuteri CCFM8631 alleviates hypercholesterolaemia caused by the paigen atherogenic diet by regulating the gut microbiota. Nutrients. (2022) 14:1272. doi: 10.3390/nu14061272, PMID: 35334930 PMC8953203

[87] Nan W Si H Yang Q Shi H Zhang T Shi Q. Effect of vitamin a supplementation on growth performance, serum biochemical parameters, intestinal immunity response and gut microbiota in American mink (Neovison vison). Animals. (2021) 11:1577. doi: 10.3390/ani11061577, PMID: 34071204 PMC8229402

[88] Mu H Zhou Q Yang R Zeng J Li X Zhang R. Naringin attenuates high fat diet induced non-alcoholic fatty liver disease and gut bacterial dysbiosis in mice. Front Microbiol. (2020) 11:585066. doi: 10.3389/fmicb.2020.585066, PMID: 33281780 PMC7691324

[89] He K Hu Y Ma H Zou Z Xiao Y Yang Y. Rhizoma Coptidis alkaloids alleviate hyperlipidemia in B6 mice by modulating gut microbiota and bile acid pathways. BBA, Mol Basis Dis. (2016) 1862:1696–709. doi: 10.1016/j.bbadis.2016.06.006, PMID: 27287254

[90] Anhê FF Nachbar RT Varin TV Trottier J Dudonné S Le Barz M. Treatment with camu camu (*Myrciaria dubia*) prevents obesity by altering the gut microbiota and increasing energy expenditure in diet-induced obese mice. Gut. (2019) 68:453–64. doi: 10.1136/gutjnl-2017-315565, PMID: 30064988

[91] Zhao R Ji Y Chen X Hu Q Zhao L. Polysaccharide from *Flammulina velutipes* attenuates markers of metabolic syndrome by modulating the gut microbiota and lipid metabolism in high fat diet-fed mice. Food Funct. (2021) 12:6964–80. doi: 10.1039/d1fo00534k, PMID: 34137411

[92] Duan R Guan X Huang K Zhang Y Li S Xia J. Flavonoids from whole-grain oat alleviated high-fat diet-induced hyperlipidemia via regulating bile acid metabolism and gut microbiota in mice. J Agric Food Chem. (2021) 69:7629–40. doi: 10.1021/acs.jafc.1c01813, PMID: 34213907

[93] Jing N Liu X Jin M Yang X Hu X Li C. Fubrick tea attenuates high-fat diet induced fat deposition and metabolic disorder by regulating gut microbiota and caffeine metabolism. Food Funct. (2020) 11:6971–86. doi: 10.1039/D0FO01282C, PMID: 32697259

[94] Zhao L Zhang Q Ma W Tian F Shen H Zhou M. A combination of quercetin and resveratrol reduces obesity in high-fat diet-fed rats by modulation of gut microbiota. Food Funct. (2017) 8:4644–56. doi: 10.1039/c7fo01383c, PMID: 29152632

[95] Chen M Liao Z Lu B Wang M Lin L Zhang S. Huang-Lian-Jie-Du-Decoction ameliorates hyperglycemia and insulin resistant in association with gut microbiota modulation. Front Microbiol. (2018) 9:2380. doi: 10.3389/fmicb.2018.02380, PMID: 30349514 PMC6186778

[96] Wei W Jiang W Tian Z Wu H Ning H Yan G. Fecal g. Streptococcus and g. Eubacterium_coprostanoligenes_group combined with sphingosine to modulate the serum dyslipidemia in high-fat diet mice. Clin Nutr. (2021) 40:4234–45. doi: 10.1016/j.clnu.2021.01.031, PMID: 33608131

[97] Vlacil AK Schuett J Ruppert V Soufi M Oberoi R Shahin K. Deficiency of Nucleotide-binding oligomerization domain-containing proteins (NOD) 1 and 2 reduces atherosclerosis. Basic Res Cardiol. (2020) 115:47. doi: 10.1007/s00395-020-0806-2, PMID: 32588196 PMC7316681

[98] Su Q. Phytochemicals in fenugreek seed prevent high fat diet induced metabolic inflammation and NAFLD via the mediation of *Akkermansia muciniphila*. Proc Nutr Soc. (2020) 79:E485. doi: 10.1017/S0029665120004334

[99] Majeed M Majeed S Nagabhushanam K Arumugam S Natarajan S Beede K. Galactomannan from Trigonella foenum-graecum L. seed: prebiotic application and its fermentation by the probiotic *Bacillus coagulans* strain MTCC 5856. Food Sci Nutr. (2018) 6:666–73. doi: 10.1002/fsn3.606, PMID: 29876118 PMC5980318

[100] Firrman J Mahalak K Bobokalonov J Liu L Lee J-J Bittinger K. Modulation of the gut microbiota structure and function by two structurally different lemon pectins. Foods. (2022) 11:3877. doi: 10.3390/foods11233877, PMID: 36496685 PMC9739951

[101] Li Q Liu W Zhang H Chen C Liu R Hou H. α-D-1, 3-glucan from Radix Puerariae thomsonii improves NAFLD by regulating the intestinal flora and metabolites. Carbohydr Polym. (2023) 299:120197. doi: 10.1016/j.carbpol.2022.120197, PMID: 36876767

[102] Shen J Zhang L Wang Y Chen Z Ma J Fang X. Beneficial actions of essential fatty acids in streptozotocin-induced type 1 diabetes mellitus. Front Nutr. (2022) 9:890227. doi: 10.3389/fnut.2022.890277, PMID: 35669071 PMC9164285

[103] Saeb A Grundmann SM Gessner DK Schuchardt S Most E Wen G. Feeding of cuticles from Tenebrio molitor larvae modulates the gut microbiota and attenuates hepatic steatosis in obese Zucker rats. Food Funct. (2022) 13:1421–36. doi: 10.1039/d1fo03920b, PMID: 35048923

[104] Lv X Wu Q Cao Y Lin Y Guo W Rao P. Ganoderic acid A from *Ganoderma lucidum* protects against alcoholic liver injury through ameliorating the lipid metabolism and modulating the intestinal microbial composition. Food Funct. (2022) 13:5820–37. doi: 10.1039/d1fo03219d, PMID: 35543349

[105] Li J Jia S Yuan C Yu B Zhang Z Zhao M. Jerusalem artichoke inulin supplementation ameliorates hepatic lipid metabolism in type 2 diabetes mellitus mice by modulating the gut microbiota and fecal metabolome. Food Funct. (2022) 13:11503–17. doi: 10.1039/d2fo02051c, PMID: 36278790

